# Enhanced hunger games search algorithm that incorporates the marine predator optimization algorithm for optimal extraction of parameters in PEM fuel cells

**DOI:** 10.1038/s41598-025-87695-0

**Published:** 2025-02-06

**Authors:** Mohamed Issa, Mohamed Abd Elaziz, Sameh I. Selem

**Affiliations:** 1 Computer Science and Information Technology Program, Egypt-Japan University of Science and Technology, New Borg El Arab, Egypt; 2 Computer and Systems Department, Faculty of Engineering, Zagazig University, Zagazig, Egypt; 3 Department of Mathematics, Faculty of Science, Zagazig University, Zagazig, Egypt; 4 Electrical power and machines Department, Faculty of Engineering, Zagazig University, Zagazig, Egypt

**Keywords:** Proton exchange membrane fuel cells, Parameters extraction, Hunger games search (HGS), Marine predator algorithm (MPA), Meta-heuristics, Electrical and electronic engineering, Computer science, Software

## Abstract

This article introduces a novel optimization approach to improve the parameter estimation of proton exchange membrane fuel cells (PEMFCs), which are critical for diverse applications but are challenging to model due to their nonlinear behavior. The proposed method, HGS-MPA, enhances the Hunger Games Search (HGS) algorithm by integrating Marine Predator Algorithm (MPA) operators, significantly boosting its exploitation capabilities and convergence rate. The effectiveness of HGS-MPA was validated on three commercial PEMFC datasets: 250-W stack, BCS 500-W, and NedStack PS6, using the Sum Squared Error (SSE) as the performance metric. Experimental results highlight that HGS-MPA achieves minimum fitness values of 0.33770, 1.31620, and 0.01174 for the respective datasets, outperforming other state-of-the-art algorithms. These findings underscore the method’s potential for accurate PEMFC parameter estimation, offering enhanced performance and reliability.

## Introduction

Proton Exchange Membrane Fuel Cells (PEMFCs) are powerful energy producers that generate clean, renewable energy^1^. PEMFCs have a range of potential real-world applications due to their unique characteristics, including high energy efficiency and environmentally friendly operation. Some notable applications include:

- Transportation: PEMFCs are promising for vehicles, especially electric cars, buses, and trucks. Their high-power density, quick start-up, and ability to operate at varying temperatures make them suitable for automotive applications.
- Portable Electronics: These fuel cells can power portable devices like laptops, smartphones, and tablets. Their compact size, lightweight, and longer runtime than traditional batteries make them appeal for extended use in remote areas or during emergencies.
- Backup Power Systems: PEMFCs can serve as reliable backup power sources for telecommunications infrastructure, data centers, and emergency power systems. Their quick start-up and ability to provide continuous power make them valuable in critical scenarios.
- Stationary Power Generation: They can be utilized for stationary power generation in homes, businesses, or remote locations where grid connectivity is limited or unreliable. PEMFCs offer a clean and efficient alternative to conventional power sources.

While PEMFCs demonstrate significant potential across various applications, achieving optimal performance depends heavily on their design and operational parameters. PEMFC’s design has multiple parameters such as the type of wind field, the volume of the catalyst, the engineering of the ribs and channels, force clamping, the width, and elements’ hydrophobicity or quality hydrophobicity, etc^2^. These parameters usually have weak and strong characteristics in cell production^3^. However, after producing a stack or a single cell and avoiding the mentioned engineering considerations, the modern alternative that assists in improving the performance is determining the optimal parameters. Numerous numerical and experimental studies are performed to examine the impacts of operating restrictions on PEMFC production^4^.

Beyond the specific context of PEMFCs, fuel cells (FCs) are generally recognized as highly efficient tools for electrochemically generating power, directly converting fuel energy into electricity. FCs provide a good environment with more productivity and favorable energy transformation based on electrochemical conversion, and they are universally recognized as a modern power source option^5^. Consequently, they have also attracted considerable consideration and fast growth for industrial stationary power production, domestic utilization, and transportation applications.

Among the various types of FCs, PEMFCs stand out due to their low operating temperature and unique advantages, such as high power density, safe and precise operation, and compact design^6^. Studies indicate that the optimal flow rate for PEMFCs ranges between 1000 and 1600 ml/min, with an ideal operating temperature of approximately 69.9 °C and an efficient range of 60–80 °C^7^.

Building on these advantages, PEMFCs are increasingly tested for vehicle utilization and power equipment that require low-heat processes and high energy frequency. Research into PEMFC operation focuses on modeling and investigating subsystems and their interactions to enhance energy quality and overall system production^8^. For example, photovoltaics merged with proton transfer membrane production of hydrogen approach is an encouraging storage system of solar energy, reforming the resting power that is sourced from photovoltaic to power transmitter with a long-term without high power frequency and pollution^9^.

Despite these advancements, the design of the polarization trajectory for specific PEMFCs presents significant challenges, particularly regarding the precise specification of various adjustment parameters^10^. Besides, it is challenging to determine the parameters within the designed model because real PEMFC production is frequently performed^11^. Indeed, the parameters estimation model is expressed as an optimization engineering problem, and the current optimizers can be employed to tackle this problem precise impersonality adjustment parameters’ values due to insufficient information, hence significant errors^12^. In the last years, various optimizers have been used in the literature to provide PEMFC model parameters^13^.

One major issue is that numerous adjustment parameters are often not considered in the PEMFC data sheets. Therefore, it is essential to identify these parameters for constructing an accurate and comprehensive model^14^. This paper aims to extract values of the PEMFC model’s optimal parameters that are not supplied in the datasheet, for example, getting polarization curves closely harmonizing the test trajectories. Knowing these parameters will help the researchers to develop a satisfactory mathematical PEMFC model quickly. The optimization rule is obliged to reduce the used fitness function representing the square difference between the calculated stack voltage and the empirical one. In this article, an improved method that combines two recent optimizers to effectively determine the PEMFCs model parameters is proposed.

Despite the significant advancements in PEMFC technology in recent years, the costs associated with this method have led to conflicts among its proponents and critics^15^. Nevertheless, the progress of PEMFC effectiveness performs a critical function in its trade perspective and achieving substantial market perception. As usual, FC production is identified as the fundamental recognition part for the end-users. Therefore, it is essential to examine the impact of operational parameters on FC production. The effectiveness of a PEMFC ultimately relies on various operational and adjustment parameters, and many investigations have focused on its development using different optimization methods. Seleem et al.^16^ proposed a novel, accurate, then-designed model of PEMFCs to estimate the problem parameters. Where the model was simplified to consist of four parameters and the integral squared error criterion was used as the objective function. Another notable innovation of this paper is the novel utilization of the equilibrium optimizer to get the PEMFCs’ unknown parameters. The high effectiveness of the proposed method is investigated under dynamic and steady-state conditions. As a result, the proposed method obtained accurate parameters for the PEMFC model. The authors of^17^ presented comparative research to analyze the influences of the adjustment of PEMFC parameters. In addition, a structural perspective and modeling of the PEMFC are performed. Moreover, this investigation intends to examine various control and clarification approaches for FC elements to find the best-enhanced performance.

Building on these optimization efforts, stochastic methods have emerged as valuable tools for navigating the search space, aiming to discover solutions that closely approach optimality^18^. Amongst stochastic methods, meta-heuristic techniques stand out as the most prevalent. They offer advantages in terms of easy implementation, simplicity, flexibility, and independence from specific problem constraints^19^. Lately, meta − heuristic algorithms have been proposed to find solutions for complex optimization problems. Recently, there has been a proposal of using meta-heuristic algorithms to address intricate optimization problems where it can find the near-optimal solution with acceptable accuracy in a reasonable time. Meta-heuristics were used in many research fields such as Bioinformatics^20,21^, Engineering Control^22–24^, parameter extraction of solar cells^25,26^, Mechanical suspensions system^27^, Cross-Docking systems^28^ and Digital Watermarking^29^.

A mathematical model representing the behavior of PEMFCs includes equations that describe the electrochemical processes within the cell and their dependencies on unknown parameters. The goal of employing meta-heuristic algorithms is to search through the parameter space and identify the combination of values that minimizes a predefined objective function, specifically the Sum Squared Error.

The objective is to develop an optimization approach that not only converges toward optimal parameter values efficiently but also addresses the computational complexities involved. By enhancing the accuracy of these parameter estimations, this research aims to improve the performance and efficiency of PEMFCs in practical applications.

In the context of PEMFC optimization, Ali et al.^30^ proposed a new PEMFC model application grey wolf optimizer (GWO) (PEMFC-GWO). Moreover, PEMFC-GWO is analyzed with other well-known methods published in the literature, and it got very competitive results. Besides, Slime Mould Algorithm (SMA) was used to optimize the selection process of PEMFC’s parameters^31^. In^32^, Coyote Optimization Algorithm (COA) was used to tune the parameters of two FC modules the 50 W stack and Ned Stack PS6 where the sum squared error criterion was used as fitness function. In^33^, the parameters of twelve PEMFCs have been estimated using Depth Information-Based Differential Evolution algorithm. Artificial Hummingbird Algorithm and Lévy flight improvement has been tested with seven PEMFC cases to find their parameters in^34^.

A developed hybrid meta-heuristic algorithm of Harris Hawk Optimization (HHO) and Atom Search Optimization (ASO) was developed for estimating the parameters of PEMFC^35^. Three commercial modules were used for the experimental tests 500 W SR-12, BCS 500-W and 250 W stack. A modified fluid search optimization algorithm was developed for estimating the precise value of PEMFC’s parameters and two commercial modules were used in the experimental tests BCS 500-W and NedStack PS6^36^. The optimal values of seven unknown parameters for five PEMFC stacks has been determined using a multi-strategy, multiple algorithms, named as the Flower Grey INFO Naked (FGIN) algorithm^37^ .In^38^, a hybrid approach of vortex search algorithm (VSA) and DE algorithms was presented for estimating the parameters of fuel cell on four modules are NedStack PS6, 250 W stack, BCS 500-W, and SR-12 PEM 500 W. An enhanced version of Arithmetic Optimization algorithm based on merging opposition operators was presented for estimating PEMFC’s parameters^39^. A multi-hybrid algorithm, known as the Kepler Red Meerkat Grey (KRMG) algorithm, has been evaluated for the parameter identification of three distinct PEMFC modules^40^.

A new hybrid approach based on Weighted Mean of Vectors and Nelder-Mead (INFONM) method is evaluated to estimate seven parameters of four available benchmark PEMFCs^41^. Also the parameters of the steady-state model for three PEMFCs using Educational Competition Optimizer (ECO) has been estimated in^42^ .In^43^, an utilization of a combination of sinusoidal parameter adaptation and L-SHADE integration in the ensemble approach was used for estimating the PEMFC’s parameters and applied on SR-12,500 W ; NedStack PS6, 6 kW; 250 W and BCS 500 W modules. In addition, another trials for the optimum estimation of PEMFC’s parameters were done such as a combination artificial bee colony with differential evolution shuffled complex^44^, shuffled multi-simplexes search algorithm^45^, Enhanced Slime Mold Algorithm^46^, heap-based optimization algorithm^47^, Barnacles Mating Optimization algorithm^48^, enhanced Archimedes optimization algorithm^49^, gradient-based optimizer^50^, Artificial Rabbits Optimization Algorithm^51^, enhanced fluid search optimization algorithm^36^, moth-flame optimization^52^, Dandelion Optimizer^53^, Jellyfish search algorithm^54^, chaotic binary shark smell optimizer^55^, an improved gorilla troops technique^56^, and hybrid sine-cosine crow search algorithm^15^.

Research trials were performed to enhance the performance of meta-heuristic algorithms rather than the hybridization between meta-heuristic ones such as ensembles methods in machine learning. It can guide this exploration by providing diverse solutions or guiding the search toward promising regions^57,58^. This diversity helps strike a balance between exploration (searching widely) and exploitation (focusing on promising areas) in metaheuristic optimization. The augmented Lagrangian approach and meta-heuristics can collaborate effectively in optimization problem-solving^59,60^. The augmented lagrangian Approach focuses on constrained optimization by combining Lagrangian relaxation with penalty functions. It aims to solve constrained optimization problems by iteratively updating Lagrange multipliers and optimizing an augmented objective function. According to the No-Free-Lunch (NFL) theorem which states that no one meta-heuristic algorithm can solve all engineering problems with the best efficient solutions.

In this research, a novel HGS^61^ was tested to estimate the PEMFC’s parameters. HGS is a recent population-based search method^61^. The HGS is produced based on the hunger-driven motions and behavioral decisions of animals. Marine Predators Algorithm (MPA) is also a population-based search method^62^. However, although HGS has efficient exploration capabilities, its exploitation capability is poor; hence a modified version of HGS was proposed in this work to enhance its exploitation. MPA is a novel meta-heuristic algorithm that has an efficient exploitation mechanism which motivates us to integrate it with HGS to gain the benefit of the exploration of HGS and exploitation of MPA. This study aims to bridge this gap by proposing an enhanced stochastic search mechanism designed to optimize the parameters crucial for the optimal functioning of PEMFCs. The existing methods often face limitations in achieving precise estimations of these parameters due to the intricate nonlinear behavior of FCs.

Therefore, this research seeks to innovate by integrating MPA operators into the HGS algorithm. This amalgamation aims to leverage the strengths of both algorithms, specifically enhancing the exploitation capabilities of HGS, to attain faster and more accurate convergence toward optimal parameter estimations. While prior research has explored various optimization techniques for FC parameter estimation, the complexities stemming from the nonlinear nature of FCs persist as a significant research gap. Existing methods often struggle to deliver precise parameter estimations, hampering the efficient utilization of PEMFCs in practical applications. This study seeks to update the existing landscape by proposing HGS-MPA, a novel hybrid algorithm that harnesses the unique characteristics of HGS and MPA to overcome the limitations faced by conventional approaches. By focusing on the convergence rate and effectiveness in achieving optimal estimations, this research aims to contribute a robust and efficient optimization methodology tailored specifically for addressing the intricacies of nonlinear behavior in PEMFCs.

The primary motivation of MPA is the general foraging approach, namely Brownian and Lévy changes in ocean predators between predators and prey. The objective function is expressed to provide the results achieved by the proposed design to present measured voltage data. Several test cases are performed and investigated to confirm the performance of the proposed method, along with several comparisons. Finally, performance measures are being conducted to prove the viability of the proposed method. Based on these results, it is concluded that the developed method found accurate PEMFC parameters compared to the well-known methods.

Building upon the extensive efforts in the literature to address the challenges of PEMFC parameter estimation PEMFCs are pivotal in advancing clean energy technologies, offering high efficiency and environmental sustainability across applications like transportation, portable electronics, and backup power systems. Despite their immense potential, accurately modeling PEMFCs remains a critical challenge due to their nonlinear behavior and dependence on numerous operational parameters. This work addresses a significant gap in optimizing PEMFC parameter estimation by proposing the hybrid HGS-MPA algorithm. The integration of the Hunger Games Search (HGS) and Marine Predator Algorithm (MPA) combines efficient exploration and exploitation capabilities, enabling precise estimations of critical parameters that conventional methods struggle to achieve. The necessity of this research lies in its ability to enhance the reliability and performance of PEMFC systems, directly contributing to their broader adoption in practical applications.

Additionally, existing optimization techniques often fall short in addressing the intricacies of nonlinear systems like PEMFCs, which hampers the development of accurate models required for real-world deployments. This research not only introduces a robust hybrid algorithm but also validates its effectiveness across multiple PEMFC modules, demonstrating superior performance in terms of accuracy, convergence, and error reduction compared to state-of-the-art methods. By improving the fidelity of PEMFC models, this study supports the design of more efficient and reliable energy systems, meeting the growing demand for sustainable energy solutions. The outcomes of this work are expected to benefit researchers and practitioners in the energy domain, further emphasizing the necessity and impact of this contribution.

The main contributions of this work are listed as follows:

- A hybrid method of HGS and MPA algorithms was proposed to enhance the exploitation phase of HGS.
- The proposed hybrid method (HGS-MPA) was used for extracting the optimal parameters of PEMFCs.
- Three PEM FC modules (250 W, BCS 500 W, and NedStack PS6) were used in the experimental tests that were collected from^54^.

The following sections in this article are organized as follows: Sect. 2 introduces the background of PEMFC and describes HGS and MPA algorithms. The proposed hybrid method (HGS-MPA) is announced in Sect. 3. Section 4 shows the experimental results including the main findings, while Sect. 5 concludes the presented work in this article along with the future extension of this effort.

## Background

### Modelling of PEMFCs

**Fig. 1:**
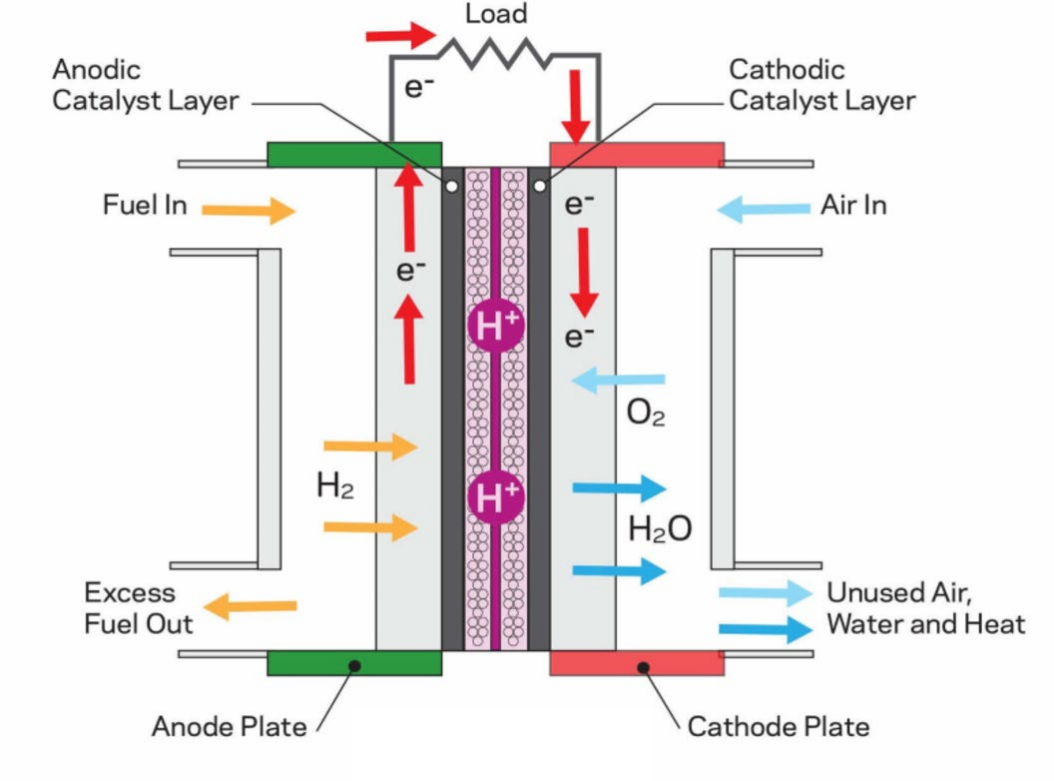
PEMFC configuration^5^.

In this section, the basic formulation of the model of PEMFC is discussed. It contains a negatively charged cathode, a positively charged anode, and an electrolyte, as depicted in Fig. 1. In the PEMFC, the hydrogen input is broken into protons and electrons using a catalyst. Also, the cathode attracts the protons, and the electrons are used to generate the output voltage by passing through the outer circuit.

Before proceeding with the mathematical model there are common assumptions used in modeling and estimating parameters for PEMFCs, which may vary depending on the study’s type as follows:

1. The PEMFC is operating in a steady-state condition, and the parameters are time-invariant and do not change significantly over time.
2. The gases involved in fuel cell operation, such as hydrogen and oxygen, behave as ideal gases. This assumption simplifies the thermodynamic modeling of the cell.
3. The PEMFC operates under isothermal conditions, meaning that the temperature is uniform throughout the cell.
4. The concentration polarization effects, such as mass transport limitations and reactant concentration gradients, are negligible or can be adequately accounted for in the model.
5. The properties of the polymer electrolyte membrane, such as proton conductivity and water sorption behavior, are constant and not influenced by the operating conditions.
6. The relationship between the system inputs, outputs, and parameters can be linearized or approximated by a linear model.

The mathematical formulation of the chemical reactions performed inside FC is given as^63^:

1 $$\:{H}_{2}\to\:2{H}^{+}+2{e}^{-}$$

2 $$\:{O}_{2}+4{e}^{-}\to\:2{O}^{-2}$$

3 $$\:{H}_{2}+\frac{1}{2}{O}_{2}\to\:{H}_{2}O+electrical\:\begin{array}{c}energy\end{array}+heat$$

where $\:{H}_{2}$ is represents molecular hydrogen $\:{H}^{+}$ represents protons and $\:{e}^{-}$ represents electrons.

In PEMFC, there are three drops for voltage that occurred, namely activation ($\:{V}_{act}$), ohmic ($\:{V}_{ohm}$) and concentration ($\:{V}_{con}$); So, the FC terminal voltage can be formulated using Eq. (4):

4 $$\:{V}_{FC}={E}_{Nernest}-{V}_{act}-{V}_{ohm}-{V}_{con}$$

In Eq. (4), $\:{E}_{Nernest}$ represents the reversible open-circuit voltage, and it is defined as^17^:

5 $$\:{E}_{Nernest}=1.229-8.5\times\:1{0}^{-4}\left(T-298.15\right)+4.385\times\:1{0}^{-5}T{ln}\left({P}_{H2}+0.5{ln}{P}_{O2}\right)$$

where $\:{P}_{O2}$ and $\:{P}_{H2}$ are the pressure of $\:{O}_{2}$ and $\:{H}_{2}$, respectively. $\:T$ denotes the cell temperature. So, the activation voltage loss ($\:{V}_{act}$) is given as^64^:

6 $$\:{V}_{act}=-[{\xi\:}_{1}+{\xi\:}_{2}\:T+{\xi\:}_{3}\:T\text{ln}\left({C}_{02}\right)+\:{\xi\:}_{4}\:T\text{ln}\left({I}_{FC}\right)]$$

In Eq. (6),$\:\:{I}_{FC}\:$denotes the FC current and $\:{\xi\:}_{1},{\xi\:}_{2},\:{\xi\:}_{3},\:{\xi\:}_{4}$ refer to the coefficients. $\:{C}_{O2}$ denotes the concentration of oxygen (mol/cm^3^), which are defined as:

7 $$\:{C}_{{O}_{2}}=\frac{{P}_{{O}_{2}}}{5.08\times\:{10}^{6}}*{e}^{\frac{498}{T}}$$

Also, the $\:{V}_{ohm}$ is results from equivalent resistance of the FC, and it is defined as:

8 $$\:{V}_{ohm}={I}_{FC}({R}_{M}+{R}_{C})$$

In Eq. (8), $\:{R}_{C}$ denotes the contact resistance and $\:{R}_{M}$ denotes the membrane resistances which define using Eq. (9):

9 $$\:{R}_{m}=\frac{{\rho\:}_{m}l}{A}$$

10 $$\:{\rho\:}_{m}=\frac{181.6\left[1+0.03\left(\frac{{I}_{FC}}{A}\right)+0.062{\left(\frac{T}{303}\right)}^{2}{\left(\frac{{I}_{FC}}{A}\right)}^{2.5}\right]}{\left[{\lambda\:}_{m}-0.634-3\left(\frac{{I}_{FC}}{A}\right)\right]{e}^{4.18*\frac{T-303}{T}}}$$

where $\:{\rho\:}_{M}$ denotes the resistivity of the membrane (Ω.cm), $\:l$ refers to the thickness of the membrane (cm), $\:A$ denotes the active area of the cell (cm^2^) and $\:{\lambda\:}_{m}$ denotes the membrane water content.

The formulation of $\:{V}_{con}$ is given as:

11 $$\:{V}_{con}=-b{ln}\left(1-\frac{\frac{{I}_{FC}}{A}}{{I}_{max}}\right)$$

In Eq. (11), $\:b$ denotes a constant and $\:{I}_{max}$ denotes the maximum current destiny (A/cm^2^). Therefore, the stack contains a series $\:n$ cells, and the stack voltage is given as:

12 $$\:{V}_{stack}=n\:{V}_{FC}=n\:({E}_{Nernest}-{V}_{act}-{V}_{ohm}-{V}_{con})$$

**Fig. 2:**
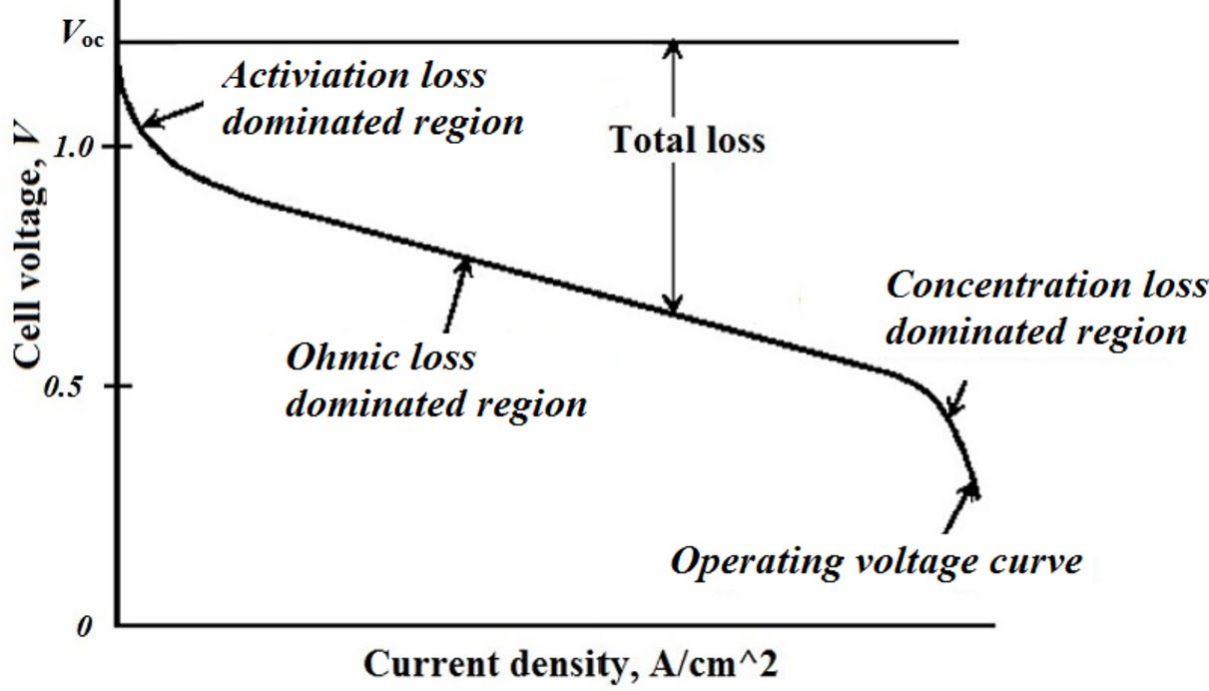
Polarization curve of PEMFC^5^.

The FC polarization curve is depicted in Fig. 2.

### Hunger games search (HGS) algorithm

HGS is a novel population-based meta-heuristic algorithm that inspired the animals’ cooperative behavior, and their activities are driven by hunger. The mathematical model of HGS consists of two stages are the approach food and the hunger rule.

The animals can cooperate socially during the foraging, and Eq. (13) represents cooperative communication and foraging behavior.

13 $$\:{{X}^{i}}_{t+1}=\:\left\{\begin{array}{c}{Game\:}_{1}\::{{X}^{i}}_{t}*\left(1+rand\right),\:\:\:\:\:\:\:\:\:\:\:\:\:\:\:\:\:{r}_{1}<l\\\:{Game\:}_{2}\::{W}_{1}*\:{X}_{b}+\:R*\:{W}_{2}*\:\left|{X}_{b}-{{X}^{i}}_{t}\right|,\:\:\:\:\:\:\:\:\:\:\:{r}_{1}>l\:,\:\:{r}_{2}>E\:\:\:\\\:{Game\:}_{3}\::\:\:{W}_{1}*\:{X}_{b}-\:R*\:{W}_{2}*\:\left|{X}_{b}-{{X}^{i}}_{t}\right|,\:\:\:\:\:\:\:{r}_{1}>l\:,\:\:{r}_{2}<E\:\end{array}\right\}$$

where $\:{r}_{1}$ and $\:{r}_{2}$ refer to random numbers belong to [0, 1]. $\:rand$ is a normal distribution random number, represents the number of iterations, $\:l$ is a constant, $\:{W}_{1}$ and $\:{W}_{2}$ are weights of the hunger which are designed according to the fact of the hunger-driven signals, $\:{X}_{b}$ denotes the location of the solution with the best fitness and $\:{{X}^{i}}_{t}$ represents the location of the agent (i) in the iteration (t).

$\:R$ represents a ranging controller that gradually reached zero and is specified as Eq. (14) while $\:E$ represents the variation control of all positions and is estimated as in Eq. (15):

14 $$\:R=\left(2\text{*}rand-1\right)\text{*}2\text{*}(1-\:\frac{t}{T}\:)$$

15 $$\:E=\:\frac{2}{{e}^{\left|F\left(i\right)-BF\right|}+\:{e}^{-\left|F\left(i\right)-BF\right|}}$$

where F(i) is the agent’s index (i) fitness and BF is the best fitness among all agents’ fitness. $\:{W}_{1}\left(i\right)$ and $\:{W}_{2}\left(i\right)$ are formulated as in Eq. (16) and Eq. (17).

16 $$\:{W}_{1}\left(i\right)=\:\left\{\begin{array}{c}hungry\:\left(i\right)*\:\frac{N}{SHungry}*\:{r}_{4}\:\:\:\:\:,\:\:\:\:\:{r}_{3}<l\\\:\:\:\:\:\:\:1,\:\:\:\:\:\:\:\:\:\:\:\:\:\:\:\:\:\:\:\:\:\:\:\:\:\:\:\:\:\:\:\:\:\:\:\:\:\:\:\:\:\:\:\:\:\:\:\:\:\:\:\:\:\:\:{r}_{3}>l\:\:\end{array}\right\}$$

17 $$\:{W}_{2}\left(i\right)=\left(1-\:{e}^{-\left|Hungry\left(i\right)-SHungry\right|}\right)\text{*}\:2\text{*}\:{r}_{5}$$

where N represents the number of agents and SHungry represents the cumulative sensation of hunger felt by all individuals, defined as the summation of their individual hungry states (sum(hungry)). $\:{r}_{3}$, $\:{r}_{4}$ and $\:{r}_{5}$ are random numbers in the range [0,1].

Hungry (i) represents the hunger of each agent and is represented as in Eq. (18):

18 $$\:Hungry\:\left(i\right)=\:\left\{\begin{array}{c}0\:,\:F\left(i\right)==BF\:\\\:Hungry\left(i\right)+H,\:\:F\left(i\right)\:!=BF\:\end{array}\right\}$$

The formula of H can be estimated based on Eq. (19) and Eq. (20):

19 $$\:TH=\:\frac{F\left(i\right)-\:BF}{WF-\:BF}\text{*}\:{r}_{6}\text{*}2\text{*}(Ub-Lb)$$

20 $$\:H=\:\left\{\begin{array}{c}LH*\left(1+r\right),\:\:\:\:TH<LH\\\:TH,\:\:\:TH>LH\end{array}\right\}$$

where$\:\:{r}_{6}\:$represents a random number in the range [0, 1], WF is the worst fitness in the iteration, LB and UB are the lower and upper boundaries of the variable in the search space in order. LH is a constant parameter tuned during experimental tests. Algorithm 1 describes the procedure of the HGS algorithm. The main merits of the HGS algorithm are concluded in two points: first, it has an efficient exploration of the search space due to ($\:{W}_{1}$ and ($\:{W}_{2}$) and avoids trapping in local optima. The second point is that due to its many parameters ($\:l,\:E$, and the random parameters ($\:{r}_{1}$ to $\:{r}_{6}$)) the diversification of the search space is efficient. However, the main drawback of HGS is the poor exploitation of the search space.

**Algorithm 1:**
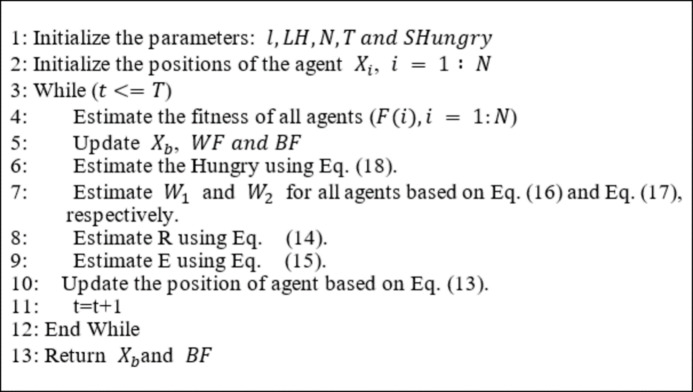
HGS algorithm.

### Marine predator algorithm (MPA)

MPA is a population-based meta-heuristic algorithm that was mimicked by the drilling strategy of ocean predators. Based on the rule of the survival of the fittest, Depredators the best suitable strategy for increasing encounter’s rate relative to their victims. However, the foraging style for many predators is the hazardous walking strategy.

Based on the concept of the survival of the fittest, skilled predators have the best skills in foraging. So, the most skilled agents are chosen and kept in an Elite matrix to explore the search space and catch victims based on their locations. According to the theory of survival of the fittest, it’s suggested that the most skilled predators in nature excel at finding food. As a result, the most adept solution is identified as a top predator and designated as part of a matrix known as Elite. Arrays within this matrix are responsible for locating and capturing prey by using information about their positions. The Elite matrix is described as in Eq. (21).

21 $$\:Elite=[\:{{X}_{1}}^{l}\:,\:{{X}_{2}}^{l}\:,\:{{X}_{3}}^{l},\:\dots\:\dots\:\dots\:.\:{{X}_{N}}^{l}]$$

Where ($\:{{X}_{i}}^{l}$) is the best fittest achieved by the agent $\:\left(i\right)$, $\:N$ represents the number of agents.

Both predator and prey are regarded as search agents, as while a predator hunts for its prey, the prey itself is seeking its own sustenance. At the conclusion of each cycle, the Elite is refreshed if a superior predator replaces the top predator in the matrix. The prey matrix is the same size as predators, which is described in Eq. (22).

22 $$\:Prey=[\:{X}_{1}\:,\:{X}_{2}\:,\:{X}_{3},\:\dots\:\dots\:\dots\:.\:{X}_{N}]$$

#### MPA’s mathematical model

Three phases for updating the prey based on MPA’s updating strategy are as follows:

##### Phase 1

The velocity ratio was high.

This phase performed the search space’s exploration and was executed in the initial matter of the iterations (t < (1/3) T). Where $\:T$ denotes the maximum number of iterations, and $\:t$ represents the current iteration. The mathematical model of this phase is described in Eq. (23).

23 $$\:{StepSize}_{i}={R}_{B}\text{*}\left({Elite}_{i}\:-\:{R}_{B}\text{*}{Prey}_{i}\right),\:i=1,\:2,\:3,\:\dots\:\dots\:.\:N$$

24 $$\:{Prey}_{i}={Prey}_{i}+P\text{*}R\text{*}\:{StepSize}_{i}$$

Where $\:{Elite}_{i}$ represent the best achieved solution by agent (i), $\:{Prey}_{i}$ represents the current solution of the agent (i), $\:{R}_{B}$ represents a normal distribution random number, $\:R\:$represents a random number that lies in the range [0, 1], $\:P$ is a constant,

##### Phase 2

The unity velocity ratio.

In this phase, during the iterations ((1/3)T < t < (2/3)T) the agents are divided into two groups (the search space’s exploration and exploitation). The first half of the population are responsible for the search space’s exploitation according to Eq. (25) and Eq. (26). While the second half of the population are responsible for exploring the search space according to Eq. (27) and Eq. (28).

25 $$\:{StepSize}_{i}={R}_{L}\text{*}\left({Elite}_{i}\:-\:{R}_{L}\text{*}{Prey}_{i}\right),\:i=1,\:2,\:3,\:\dots\:\dots\:.\:N/2$$

26 $$\:{Prey}_{i}={Prey}_{i}+P\text{*}R\text{*}\:{StepSize}_{i}$$

27 $$\:{StepSize}_{i}={R}_{B}\text{*}\left({Elite}_{i}\:-\:{R}_{B}\text{*}{Prey}_{i}\right),\:i=\frac{N}{2},\:\dots\:\dots\:\dots\:.n$$

28 $$\:{Prey}_{i}={Prey}_{i}+P\text{*}CF\text{*}\:{StepSize}_{i}$$

$\:P$ is a constant, $\:{R}_{B}$ represents a normal distribution random number, $\:{R}_{L}$ denotes a vector of random numbers generated based on the Levy distribution, representing Levy’s movement. While the adaptive parameter ($\:CF$) is modeled as in Eq. (29)

29 $$\:CF=\:{\left(1-\frac{t}{T}\right)}^{\frac{2t}{T}}$$

##### Phase 3

The velocity ratio was assumed to be lower.

This phase performs more intensive exploitation for iterations (t > (2/3) T) and the updating mechanism as described in Eq. (30).

30 $$\:{StepSize}_{i}={R}_{L}\text{*}\left({Elite}_{i}\:-\:{R}_{L}\text{*}{Prey}_{i}\right),\:i=1,\:2,\:3,\:\dots\:\dots\:.\:N$$

31 $$\:{Prey}_{i}={Prey}_{i}+P\text{*}CF\text{*}\:{StepSize}_{i}$$

##### Fish aggregating devices (FAD) effect

FAD simulates the environmental issues around the marine predator, which simulates a local optimum. A long jump is needed to avoid trapping in local optima, performed as in Eq. (32).

32 $$\:{Prey}_{i}=\:\left\{\begin{array}{c}{Prey}_{i}+CF\:[{X}_{min}+R*\left({X}_{max}-\:{X}_{min}\right]\:\:\:\:\:\:\:\:\:\:\:\:\:\:\:\:\:\:\:\:\:\:if\:r\le\:\:FAD\\\:{Prey}_{i}+[\:FAD\left(1-r\right)+r)\left]*\right({Prey}_{r1}-\:{Prey}_{r2})\:\:\:\:\:\:\:\:\:\:if\:r>FAD\end{array}\right\}$$

where r is a random number in the range [0, 1], $\:FAD$ is a fixed value, $\:{X}_{max}$ and $\:{X}_{min}$ represent the upper and lower bounds, $\:{Prey}_{r1}$ and $\:{Prey}_{r2}$ are random solutions. The procedural steps of MPA were described in Algorithm 2.

**Algorithm 2:**
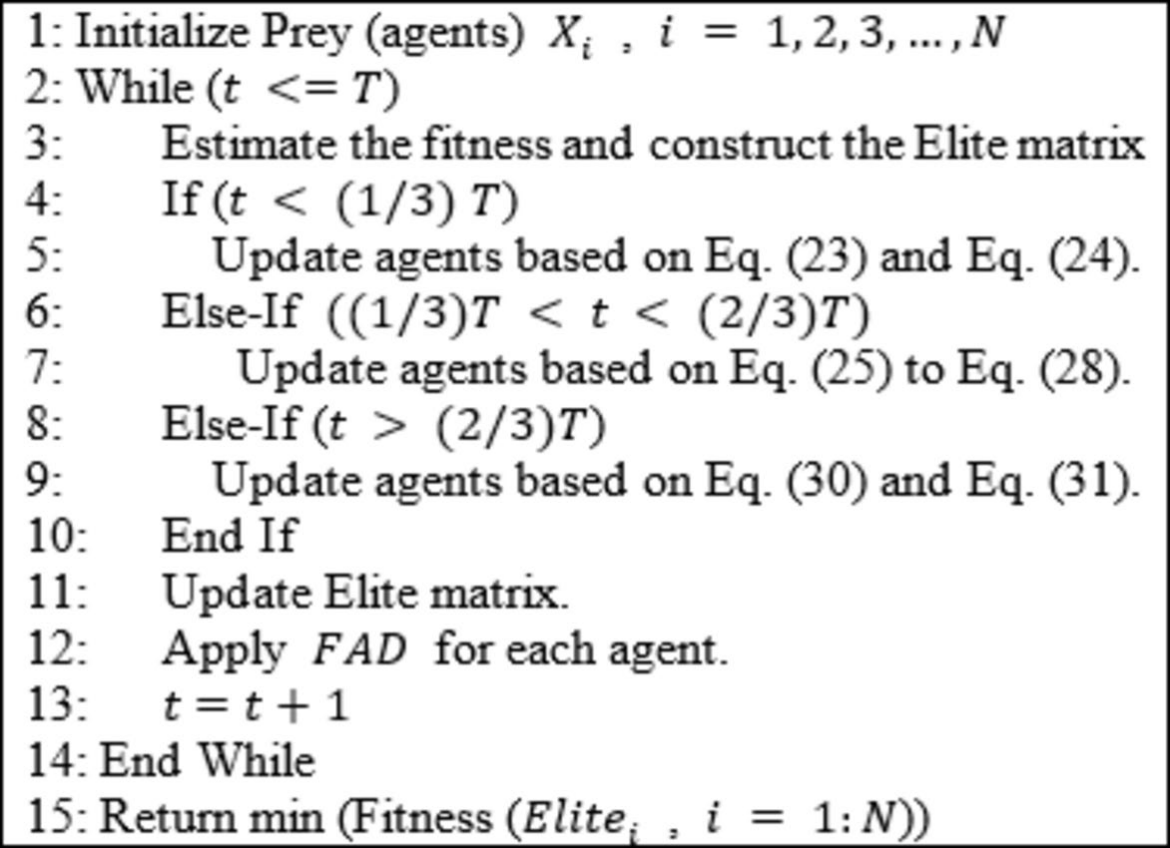
MPA algorithm.

MPA has been chosen to enhance the exploitation of the HGS algorithm due to the following reasons:

1. It avoids trapping in local optima due to applying FAD.
2. It has an efficient exploitation scheme better than exploration since it has a good memory for successful foraging.

## Proposed HGS-MPA

In this section, the hybridization mechanism between HGS and MPA is discussed. HGS has better diversification since it has many random variables besides the weights of hungry ($\:{W}_{1}$) and ($\:{W}_{2}$) increase its immunity for avoiding trapping in local optima. Unfortunately, HGS’ exploitation needs to be enhanced, which is the focus of this work. An efficient exploitation strategy of another algorithm can be added to HGS to enhance its intensification of the search space. MPA’s exploitation capability is efficient due to the memory updating of the skilled predators; besides, it enhances the avoidance of trapping in local optima due to applying FAD. Algorithm 3 describes the merging of two techniques.

**Fig. 3:**
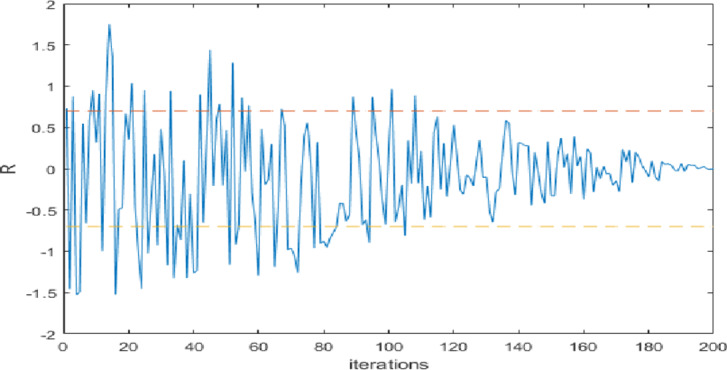
The function of ($\:R$) in terms of iterations^61^.

In line (1), the parameters of the developed algorithm are initialized with fixed values, while the agents are initialized with random values in the range ($\:{X}_{min}$ ,$\:\:{X}_{max}$) as in line (2). The while loop starts execution in line (3), and the lines (4–17) are executed for (T) iterations. In line (4), the parameter (R) is updated based on Eq. (14), and the fitness of all search agents is estimated according to the objective function as inline (5). The skilled predators are updated in the Elite matrix in line (6), while the best solution ($\:{X}_{b}$), the best fitness ($\:BF$), and the worst fitness ($\:WF$) are updated in line (7). Line (8) determines the execution of HGS (exploration) or MPA (exploitation) updating strategies based on the value of ($\:R$) where if the absolute value of ($\:R$) is larger than (0.7), then execute HGS else exploit the search space based on MPA’s updating strategy. As shown in Fig. 3, for the initial iterations, the algorithms tend for more exploration based on the HGS updating strategy, while for iterations near the final, the developed algorithm tends to exploit the search space using the exploitation updating strategy of MPA. The value (0.7) of $\:R$ was chosen due to experimental tests that choose the best values of R that produce the best result.

**Algorithm 3:**
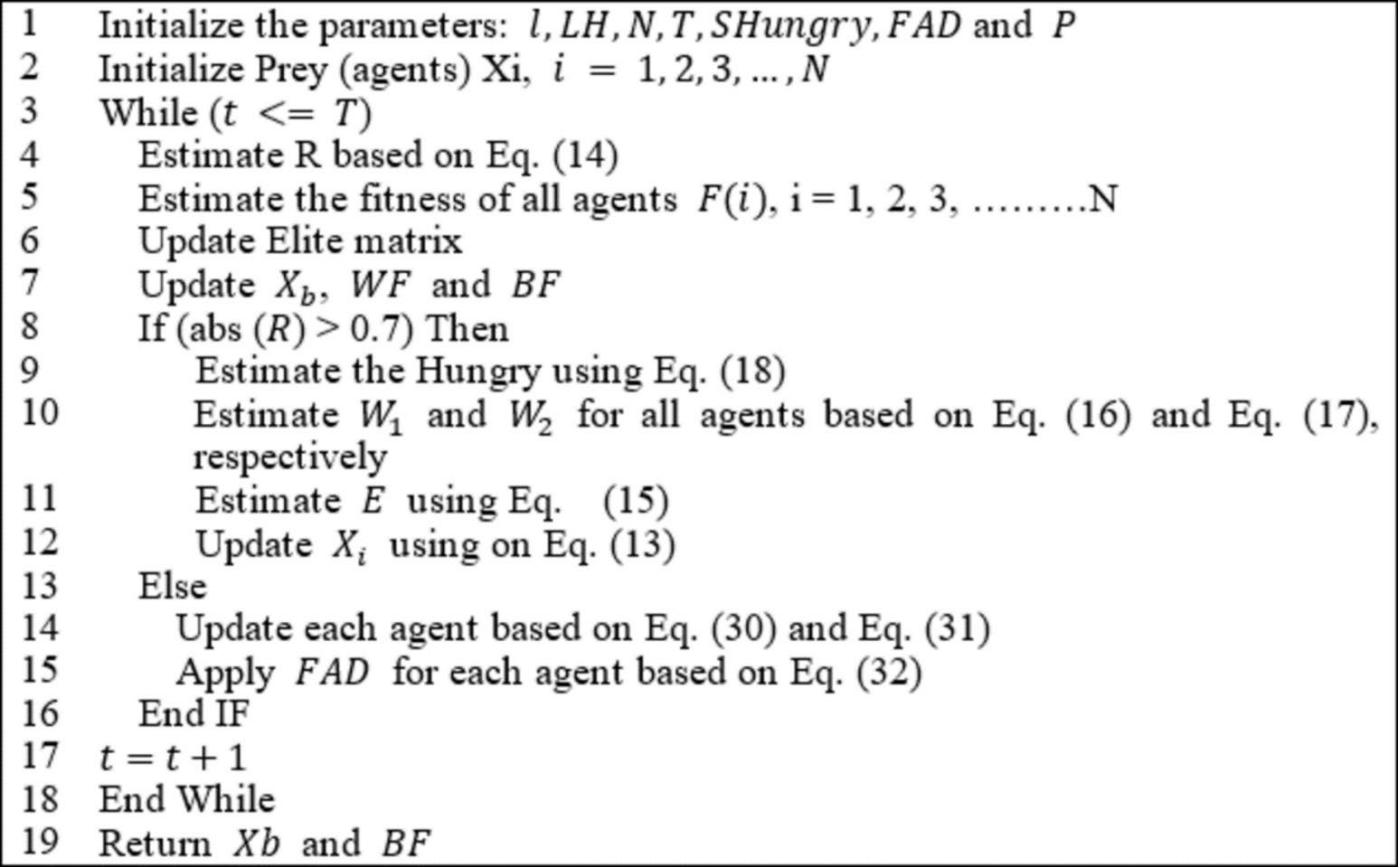
HGS-MPA algorithm.

Lines (9–11) are executed on all agents where line (9) estimates the hungry of agents based on Eq. (10) while W_1_ and W_2_ are estimated based on Eq. (16) and Eq. (17) in order as in line (10). The parameter ($\:E$) is estimated based on Eq. (15) as in line (11), and the updating of agent’s positions is updated based on the updating strategy of the HGS algorithm.

**Fig. 4:**
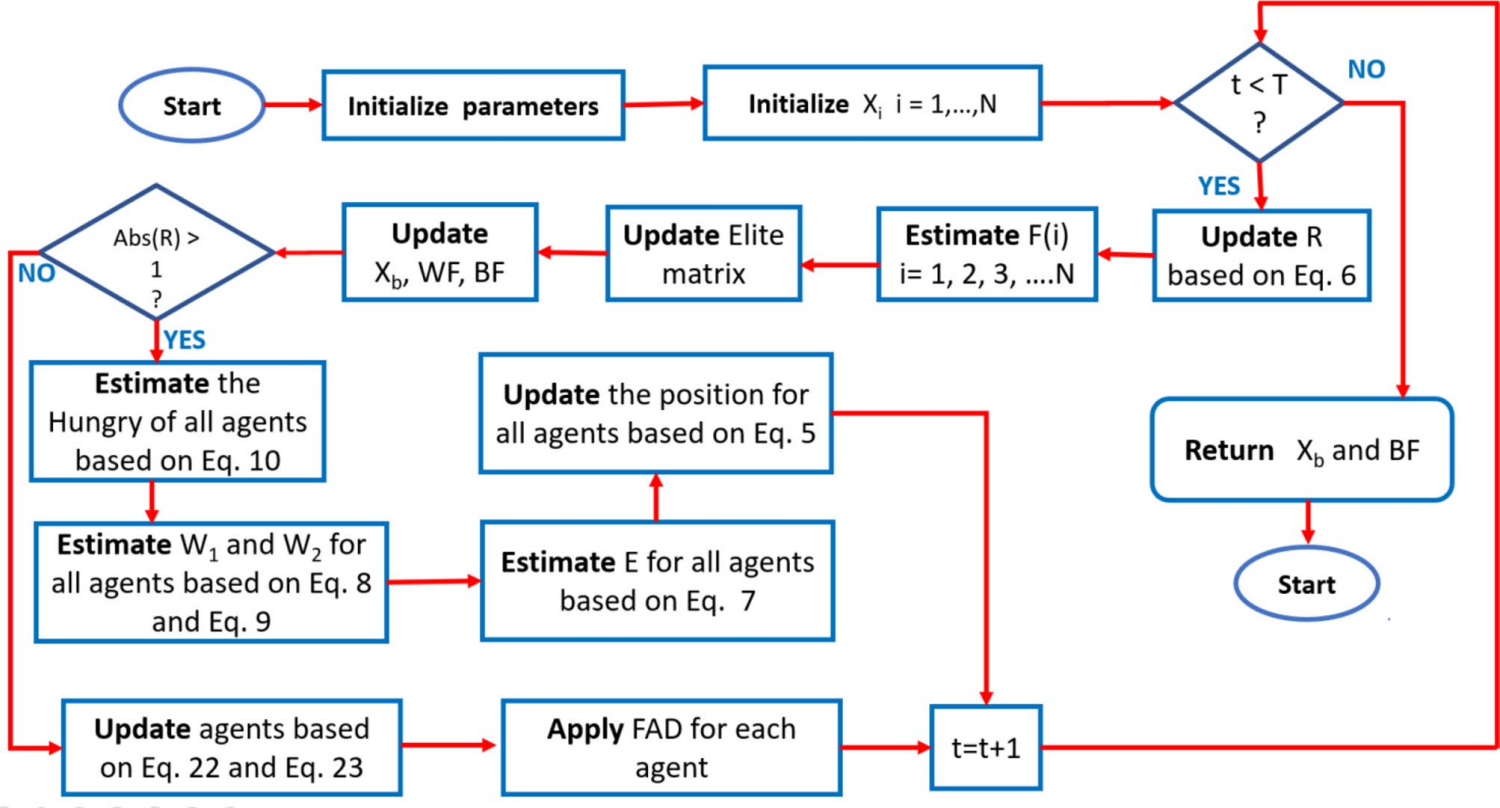
Steps of HGS-MPA for PEMFC.

Line (14) updates the agents’ positions based on Eq. (30) and Eq. (31) using the exploitation updating strategy of MPA. In line (15), the $\:FAD$ is applied for all agents to avoid trapping in local optima. The output is released in line (19), which is represented in the best solution ($\:{X}_{b}$) and the best fitness ($\:BF$). Figure 4 presents the procedure of the flowchart of the developed HGS-MPA algorithm.

The time complexity of the HGS-MPA algorithm is $\:O\left(N\:\text{*}\:T\:\text{*}\:max\:\right({C}_{HGS},\:{C}_{MPA}\left)\right)$ where $\:N$ represents the number of agents, $\:T$ is the number of iterations, C_HGS_ is the cost of updating one agent based on the HGS’ updating strategy (lines (9–11)) and C_MPA_ is the cost of updating one agent based on the MPA’s updating strategy (lines (14–15)).

## Numerical analysis

The performance of HGS-MPA was evaluated for estimating the PEMFC’s parameters on a set of three datasets, namely BCS 500-W^2,33,34^, 250-W^33,34^ and NedStack PS6^33^ types are used in experimental tests with specifications, as illustrated in Table 1. Fifteen measurements datasets for 250 W module, eighteen measurements datasets for BCS 500 W and twenty-nine measurements datasets for NedStack PS6 are used to validate the HGS-MPA cropped optimal PEMFCs stack parameters. MATLAB 2018 software was used for running experiments on a computer machine with specifications such as Core i5-4210U (1.70 GHz) and 8 GB RAM.

Table 2 shows the two domains of the variables used in this study^15^. The results of the developed method are compared with a set of meta-heuristic techniques, including HHO, Political optimizer (PO), shuffled multi-simplexes search algorithm (SMS), interior search algorithm (ISA), vortex search algorithm (VSA), grey-wolf optimization (GWO), Sine-cosine algorithm (SCA), and the traditional HGS. The parameter setting for these algorithms is set according to the original implementation. The standard parameters between the algorithms, such as the number of iterations and the total number of solutions, are 500 and 50, respectively.

Table 3 presents a comparative analysis of the developed HGS-MPA model against various other models using three distinct datasets. The HGS-MPA model consistently demonstrates superior performance across all datasets, as evidenced by the consistently lower SSE values compared to the other models. This indicates that the HGS-MPA model achieves a better fit for the experimental data, suggesting that it more accurately captures the underlying dynamics of the photovoltaic system. Notably, the HGS-MPA model excels in fitting the 250 W and 500 W datasets, achieving the lowest SSE values among all models. This highlights the HGS-MPA’s robustness and adaptability across different system configurations and operating conditions. Furthermore, the table reveals that the HGS-MPA model extracts parameter values that are closer to the expected ranges, suggesting a higher degree of accuracy and reliability in model parameter estimation. In summary, the results presented in Table 3 strongly support the efficacy of the HGS-MPA model as a promising tool for accurate PEMFC modeling and performance prediction.

**Table 1:** PEMFC stack specifications.

	$\:cell{s}_{nb}$	$\:L\left(\mu\:m\right)$	$\:{A}_{m}\left(c{m}^{2}\right)$	$\:{CD}_{max}\left(\frac{mA}{c{m}^{2}}\right)$	$\:T{M}_{c}\left(K\right)$	$\:P{R}_{H2}\left(atm\right)$	$\:P{R}_{O2}\left(atm\right)$
BCS 500-W^2,33,34^	32	178	64	469	333	1	0.2095
250-W^33,34^	24	127	27	860	343	1	1
PS6^33^	65	178	240	1125	343	(0.5-5) 1	(0.5-5) 1

**Table 2:** Two parameter ranges of PEMFC parameters.

		ξ_1_	ξ_2_	ξ_3_	ξ_4_	λ	R_c_ (Ω)	b (V)
Range 1	Lower limit	-1.1997	0.80E-3	3.60E-5	-26.00E-5	13	1.0E-4	0.0136
	Upper limit	-0.8532	6.00E-3	9.80E-5	-9.54E-5	23	8.0E-4	0.5000

**Table 3:** The estimated PEMFC’s near-optimal parameters for range 1.

		ξ_1_	ξ_2_	ξ_3_	ξ_4_	λ	*R*_c_	b	SSE
BCS 500 W	HGS-MPA	-0.94490	3.30E-03	7.59E-05	-7.39E-05	18.78489	4.90E-04	0.01600	**1.31620**
	HGS	-0.95100	3.39E-03	7.80E-05	-9.21E-05	14.00000	1.40E-04	0.01707	2.11380
	VCA	-0.94820	3.33E-03	7.63E-05	-7.87E-05	19.19689	4.00E-04	0.02483	1.54930
	SCA	-0.94750	3.31E-03	7.67E-05	-7.19E-05	19.56995	4.10E-04	0.02905	8.72860
	GWO	-0.94770	3.33E-03	7.63E-05	-8.07E-05	18.59923	3.20E-04	0.03018	1.91880
	MPA	-0.94790	3.32E-03	7.59E-05	-7.76E-05	16.77981	3.80E-04	0.01602	1.35240
250 W	HGS-MPA	-0.94450	3.18E-03	7.43E-05	-18.0E-05	15.50566	1.10E-04	0.01600	**0.33770**
	HGS	-0.94400	3.23E-03	7.80E-05	-17.0E-05	14.97517	1.00E-04	0.01600	0.34700
	VCA	-0.94850	3.23E-03	7.67E-05	-18.0E-05	18.70992	4.80E-04	0.01790	0.37080
	SCA	-0.94870	3.23E-03	7.69E-05	-18.0E-05	18.39506	2.80E-04	0.01846	0.54636
	GWO	-0.94780	3.22E-03	7.60E-05	-18.0E-05	18.23128	3.50E-04	0.01822	0.36809
	MPA	-0.99750	2.40E-03	3.60E-05	-15.5E-05	23.99000	1.00E-04	0.05590	0.59400
	HHO	-1.10972	3.45E-03	8.32E-05	-15.2E-05	22.94500	3.83E-04	0.05420	0.64577
	PO	-1.08933	3.62E-03	6.18E-05	-15.1E-05	23.99900	5.00E-04	0.05470	0.64421
NedStack PS6	HGS-MPA	-0.94820	3.21E-03	7.60E-05	-19.0 E-05	20.98452	1.10E-04	0.01614	**0.01174**
	HGS	-0.94540	3.23E-03	7.80E-05	-19.0 E-05	19.27337	1.00E-04	0.01600	0.04620
	VCA	-0.94770	3.22E-03	7.65E-05	-18.0E-05	20.31023	3.70E-04	0.01610	0.10337
	SCA	-0.94760	3.21E-03	7.54E-05	-19.0E-05	20.81781	1.80E-04	0.01819	0.66195
	GWO	-0.94930	3.21E-03	7.58E-05	-19.0E-05	21.65000	2.90E-04	0.01634	0.03821
	MPA	-0.98640	2.61E-03	3.60E-05	-19.3E-05	20.81670	1.00E-04	0.01156	0.01156
	JSA	-1.11630	3.78E-03	8.03E-05	-9.54E-05	13.46500	1.00E-04	0.01360	2.14570
	ISA	-0.85530	2.42E-03	3.72E-05	-9.00E-05	13.49700	1.00E-04	0.01360	2.16720
	SMS	-0.95250	2.91E-03	5.18E-05	-9.54E-05	12.57400	1.00E-04	0.01360	2.06550

Significant values are in bold.

**Fig. 5:**
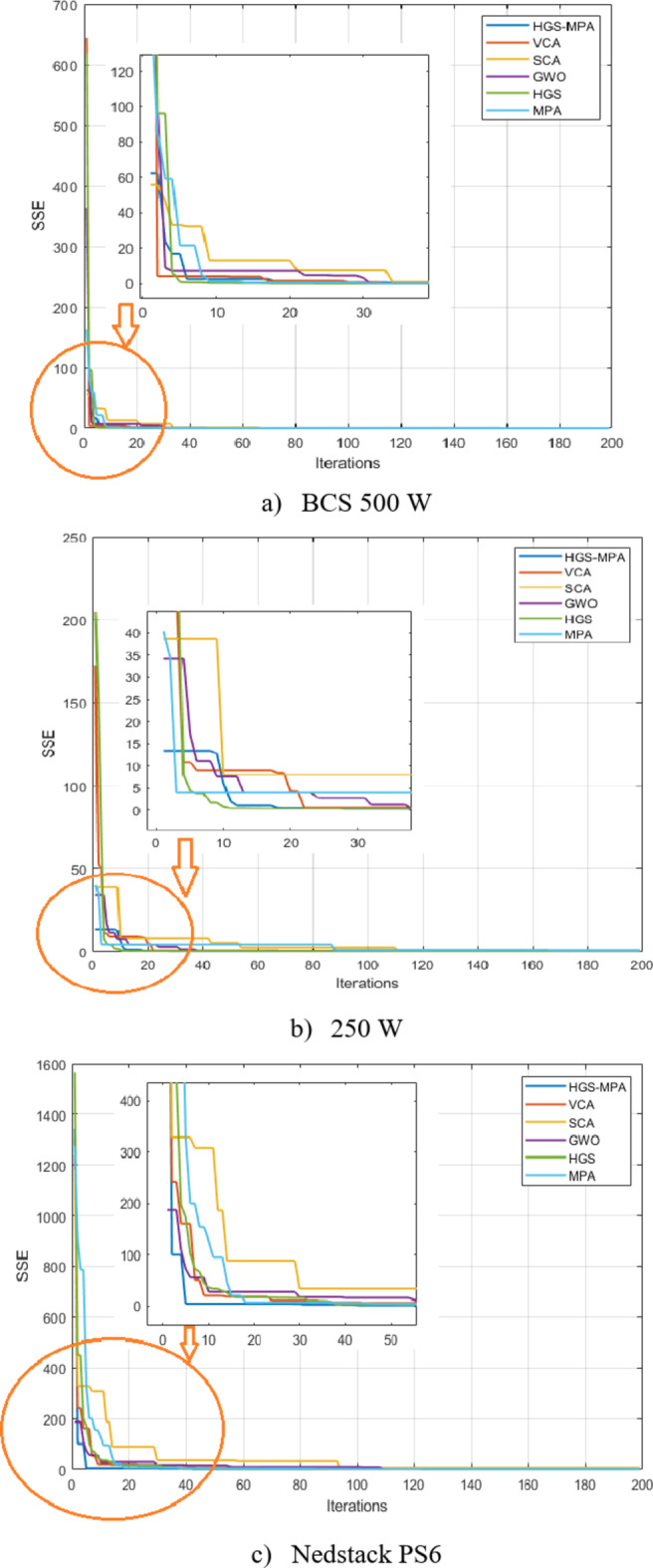
Convergence of competitive algorithm overall the tested datasets.

Figure 5 shows the convergence of HGS-MPA and other models. This figure illustrates the convergence behavior of the HGS-MPA model in comparison to other optimization algorithms (VCA, SCA, GWO, HGS, MPA) for the modules. A significant highlight is that the HGS-MPA demonstrates a noticeably faster convergence rate for the NedStack module compared to the other algorithms. This is evident from the steeper initial drop in the SSE curve for HGS-MPA. This rapid convergence suggests that HGS-MPA can efficiently refine its solution and reach a near-optimal state within fewer iterations. Competitive convergence for other datasets (250 W and 500 W), the HGS-MPA exhibits a convergence rate that is competitive with the MPA and other algorithms. While it might not be the absolute fastest in these cases, it still demonstrates a reasonable convergence speed. Overall, Fig. 5 provides strong evidence that the HGS-MPA model is an effective optimization approach, particularly for the NedStack module where it shows superior convergence characteristics.

**Fig. 6:**
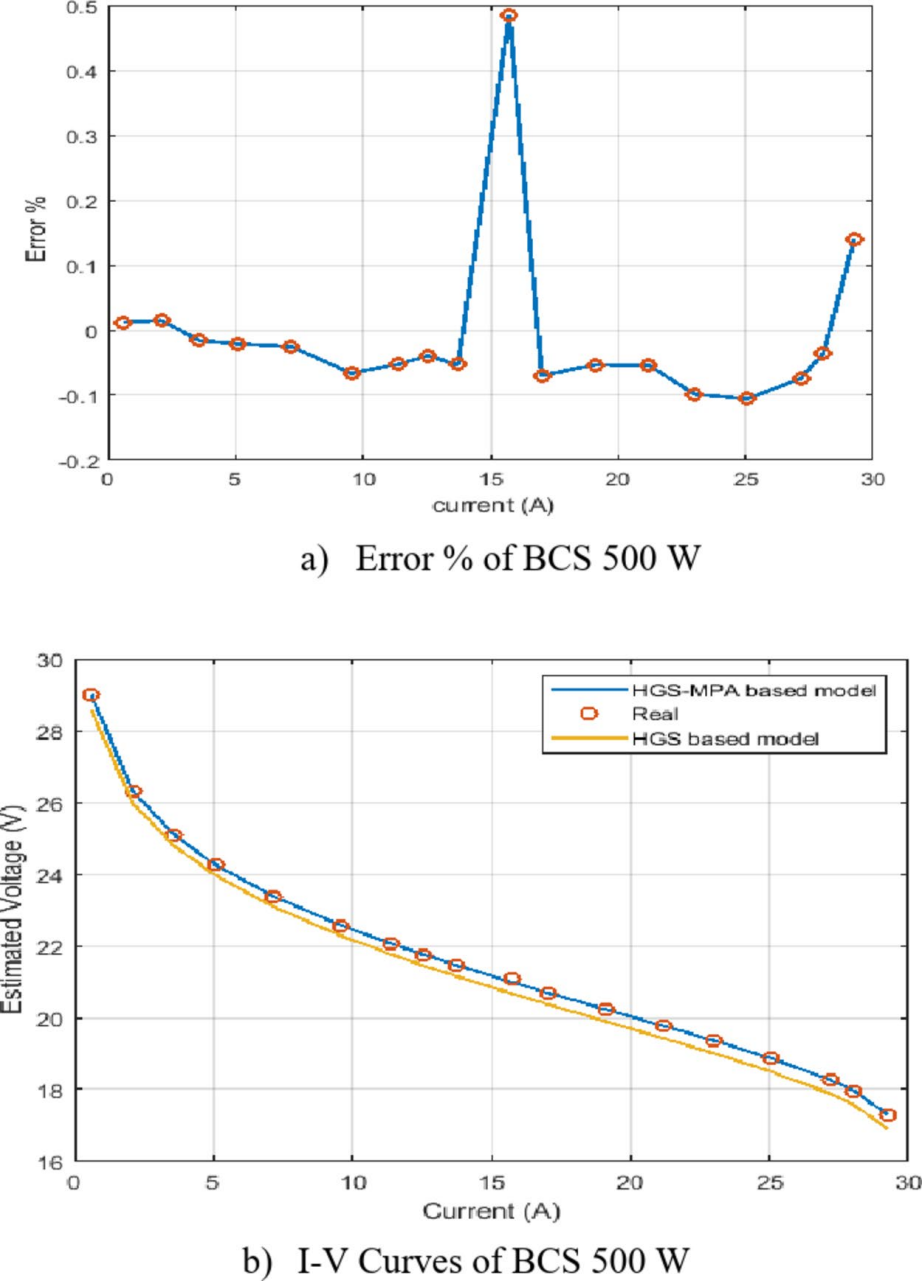
Percentage error and I-V curves of the developed HGS-MPA for the BCS 500 W module.

In addition, the curves of measured I/V polarization and the percentage errors of the voltage are depicted in Fig. 6. The percentage error was estimated as in Eq. (33):

33 $$\:Error\:\%=\:\frac{{V}_{est}-\:{V}_{exp}}{{V}_{exp}}*100$$

Figure 6 provides insights into the performance of the HGS-MPA model in predicting the I-V characteristics of a 500 W stack.

- Figure 6 (a) Error % of BCS 500 W:

The error percentage plot shows the deviation between the estimated voltage by the HGS-MPA model and the measured voltage. The error is generally low, indicating good agreement between the model and the real system’s behavior. There is a noticeable spike in the error around 15 A. This could be due to various factors, such as noise measurement, variations in environmental conditions, or limitations in the model’s accuracy in that specific region.

b) Figure 6 (b) I-V Curves of BCS 500 W:

The I-V curves compare the estimated voltage from the HGS-MPA model (blue curve) to the real measured voltage (orange curve). The curves overlap closely, suggesting that the HGS-MPA model accurately captures the overall I-V characteristics of the 500 W system. The slight deviations between the curves are consistent with the error percentage plot, with the largest deviation occurring around 15 A.

Overall, Fig. 6 demonstrates that the HGS-MPA model provides a good approximation of the I-V characteristics of the 500 W stack. The low error percentage and close agreement between the estimated and measured I-V curves suggest that the model can be effectively used for system analysis and performance prediction.

**Fig. 7:**
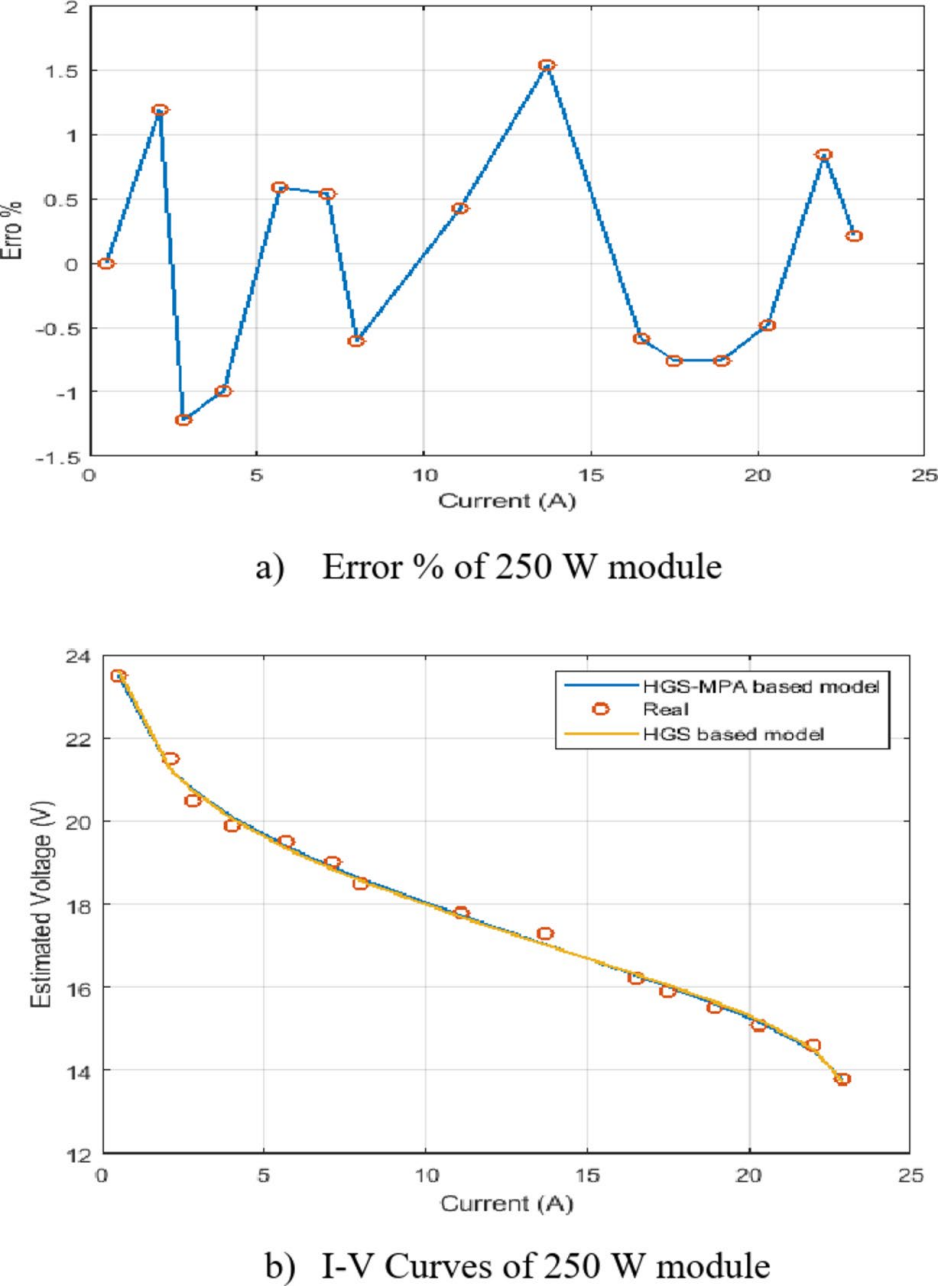
Percentage error and I-V curves of the developed HGS-MPA for 250 W module.

Figure 7 demonstrates the HGS-MPA model’s ability to provide a good approximation of the I-V characteristics for the 250 W photovoltaic system. The low error percentage and close agreement between the estimated and measured I-V curves indicate that the model can be a valuable tool for system analysis and performance prediction.

a) Fig. 7 (a) Error % of 250 W Module:

The error percentage plot in Fig. 7 (a) visualizes the deviation between the voltage estimated by the HGS-MPA model and the actual measured voltage for a 250 W photovoltaic module. The error is generally low across most current values, indicating a good agreement between the model’s predictions and the real-world system behavior. However, there is a noticeable spike in the error around 15 Amps. This could be attributed to several factors, such as measurement noise, fluctuations in environmental conditions, or limitations in the model’s accuracy within this specific current range.

b) Fig. 7 (b) I-V Curves of 250 W Module:

Figure 7 (b) presents a comparison of the estimated voltage from the HGS-MPA model (blue curve) with the actual measured voltage (orange curve) for the 250 W module. The close overlap between the two curves strongly suggests that the HGS-MPA model effectively captures the overall I-V characteristics of the module. Minor deviations between the curves are observed, aligning with the error percentage plot, with the largest deviation occurring around 15 Amps, as seen in Fig. 7 (a).

Fifteen measurement datasets for the 250 W module, eighteen measurement datasets for BCS 500 W, and twenty-nine measurement datasets for NedStack PS6 are used to validate the HGS-MPA cropped.

Optimal PEMFCs stack parameters. Table 4 presents a comparison of the measured voltage versus the estimated voltage obtained by the HGS-MPA model for three different PV modules: 250 W, BCS 500 W, and NedStack PS6. The table demonstrates a good match between the measured and estimated voltages across all three modules. This is evidenced by the generally small differences between Vexp and Vout. The HGS-MPA model appears to be accurate in estimating the voltage output for all three modules. The close agreement between Vexp and Vout suggests that the model captures the underlying physics of PEMFC modules effectively.

**Fig. 8:**
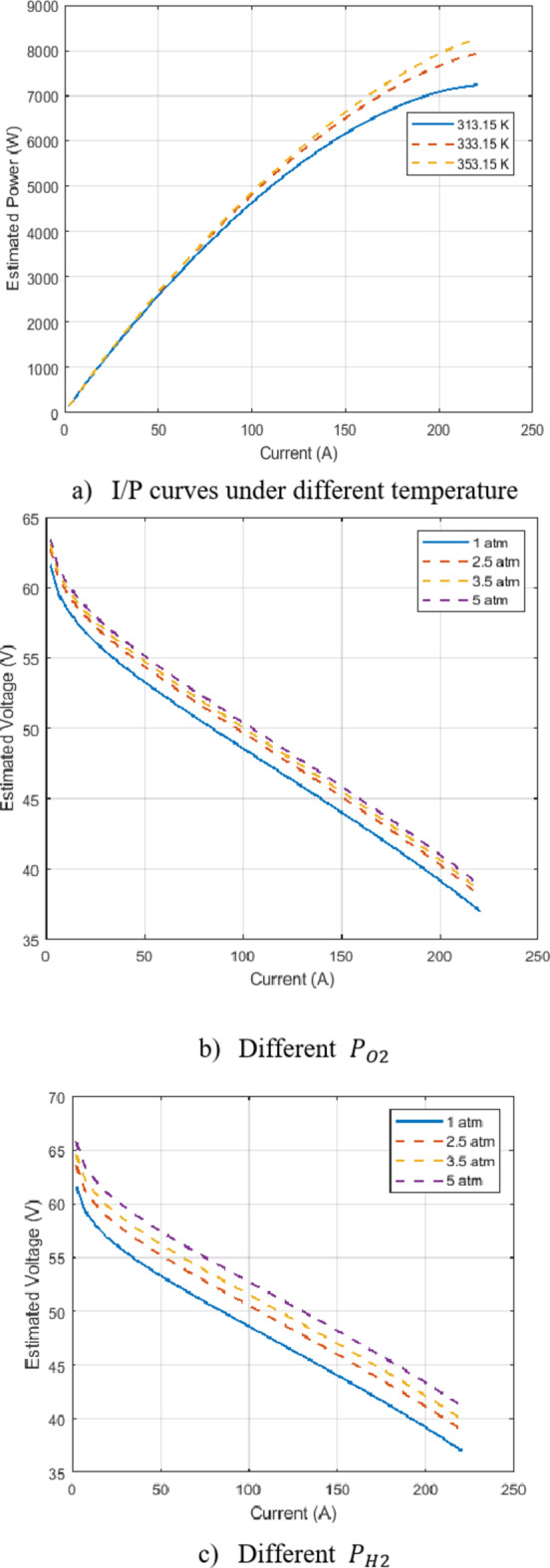
Results of BCS 500 W stack using the HGS-MPA.

Figure 8 shows the influence of using different temperatures on the efficiency of the PEMFCs stack preserving the pressures fixed at $\:{P}_{O2}$ and $\:{P}_{H2}$. Figure 8 (a) shows the effect of three different temperatures on I/P curves, respectively. By operating at higher temperatures within a suitable range, it is possible to achieve higher power output and potentially improved efficiency. However, careful consideration should be given to the trade-offs between temperature and other factors like fuel crossover and degradation to ensure optimal overall performance and durability. As the temperature increases from 313.15 K (40 °C) to 353.15 K (80 °C), the overall power output of the PEMFC stack increases. This is evident from the upward shift of the I/P curves with increasing temperature.

The effect of different regulating pressures of oxygen is depicted in Fig. 8 (b) and this conducted as $\:T{M}_{c}$=70^o^ C and $\:{P}_{H2}=1$ atm. This figure shows the influence of different oxygen-regulating pressures (1 atm, 2.5 atm, 3.5 atm, and 5 atm) on the voltage output of a PEMFC stack. The analysis is conducted at a constant cell temperature of 70 °C and a hydrogen pressure of 1 atm. As the oxygen pressure increases from 1 atm to 5 atm, the overall voltage output of the PEMFC stack also increases. This is evident from the upward shift of the voltage curves with increasing oxygen pressure. The voltage curves exhibit a characteristic decreasing trend with increasing current. This is typical behavior for PEMFCs, where the voltage drops due to various losses (activation, ohmic, and concentration) as the current density increases.

As well as the $\:{P}_{H2}$ is the variant with a setting $\:{P}_{O2}=1.5$ atm and $\:T{M}_{c}$=70^o^ C as shown in Fig. 8 (c). Figure 8 (c) demonstrates the combined influence of temperature and oxygen pressure on the voltage output of a PEMFC stack. The analysis is conducted at a constant hydrogen pressure of 1 atm and varying oxygen pressures (1 atm, 2.5 atm, 3.5 atm, and 5 atm) at a fixed cell temperature of 70 °C. As shown in the figure, increasing the oxygen pressure leads to a higher voltage output for each temperature condition. The voltage curves shift upwards as the oxygen pressure increases.

**Table 4:** Estimated voltage obtained by HGS-MPA.

250 W module			BCS 500 W			NedStack PS6		
$\:{I}_{fc}\left(A\right)$	$\:{V}_{exp}\left(V\right)$	$\:{V}_{est}\left(V\right)$	$\:{I}_{fc}\left(A\right)$	$\:{V}_{exp}\left(V\right)$	$\:{V}_{est}\left(V\right)$	$\:{I}_{fc}\left(A\right)$	$\:{V}_{exp}\left(V\right)$	$\:{V}_{est}\left(V\right)$
0.5	23.5	23.50020	0.60	29.00	28.99491	2.25	61.64	61.62431
2.1	21.5	21.24456	2.10	26.31	26.30542	6.75	59.57	59.51630
2.8	20.5	20.74999	3.58	25.09	25.09353	9.00	58.94	58.89353
4.0	19.9	20.09752	5.08	24.25	24.25476	15.75	57.54	57.52392
5.7	19.5	19.38583	7.17	23.37	23.37554	20.25	56.80	56.81007
7.1	19.0	18.89763	9.55	22.57	22.58456	24.75	56.13	56.17839
8.0	18.5	18.61193	11.35	22.06	22.07108	31.50	55.23	55.32689
11.1	17.8	17.72396	12.54	21.75	21.75807	36.00	54.66	54.80230
13.7	17.3	17.03436	13.73	21.45	21.46072	45.00	53.61	53.82090
16.5	16.2	16.29435	15.73	21.09	20.98695	51.75	52.86	53.12607
17.5	15.9	16.02056	17.02	20.68	20.69356	67.50	51.91	51.58905
18.9	15.5	15.61772	19.11	20.22	20.22979	72.00	51.22	51.16444
20.3	15.1	15.17245	21.20	19.76	19.76955	90.00	49.66	49.50307
22.0	14.6	14.47709	23.00	19.36	19.36452	99.00	49.00	48.68576
22.9	13.8	13.77063	25.08	18.86	18.86494	105.80	48.15	48.07094
			27.17	18.27	18.27340	110.30	47.52	47.66463
			28.06	17.95	17.95226	117.00	47.10	47.05967
			29.26	17.30	17.29270	126.00	46.48	46.24541
						135.00	45.66	45.42720
						141.80	44.85	44.80507
						150.80	44.24	43.97478
						162.00	42.45	42.92759
						171.00	41.66	42.07218
						182.30	40.68	40.97645
						189.00	40.09	40.31318
						195.80	39.51	39.62785
						204.80	38.73	38.69874
						211.50	38.15	37.98775
						220.50	37.38	37.00069

Table 5 presents statistical measures for the SSE (Sum of Squared Errors) values obtained by different optimization algorithms (HGS-MPA, HGS, VCA, SCA, GWO) when applied to three different PEMFC modules: BCS 500 W, 250 W, and NedStack PS6. The statistics include mean, standard deviation, best SSE, and worst SSE. The statement correctly points out that the HGS-MPA algorithm generally provides better results compared to the other methods. For all three modules, the HGS-MPA consistently shows the lowest mean SSE value. This indicates that, on average, HGS-MPA produces more accurate parameter estimates with lower prediction errors. The standard deviation of SSE values for HGS-MPA is significantly lower than other methods. This suggests that HGS-MPA provides more consistent and reliable results with less variability across different runs. Hence, Table 5 and the accompanying analysis provide compelling evidence that the HGS-MPA algorithm outperforms other optimization methods in estimating PEMFC parameters. Its statistical measures demonstrate superior accuracy, consistency, and reliability, making it a promising tool for PEMFC modeling and performance prediction.

**Table 5:** The statistical analysis of the estimated parameters of the three modules.

Dataset	Algorithm	Std	Worst	Best	Mean
BCS 500 W	HGS-MPA	**0.00710**	**1.3361**	**1.3134**	**1.31620**
	HGS	0.93410	4.5797	1.3265	2.11380
	VCA	0.27782	2.477065	1.324782	1.54930
	SCA	3.33839	19.31522	5.150285	8.72860
	GWO	0.59551	3.806807	1.357709	1.91880
250 W	HGS-MPA	**0.00001**	**0.3377**	**0.3377**	**0.33770**
	HGS	0.01870	0.3985	0.3377	0.34700
	VCA	0.04041	0.449532	0.337841	0.37080
	SCA	0.15546	0.955228	0.422466	0.54636
	GWO	0.02118	0.408939	0.341183	0.36809
NedStack PS6	HGS-MPA	**1.25E-05**	**0.01174**	**0.011701**	**0.01174**
	HGS	4.75E-02	0.145	0.0118	0.04620
	VCA	0.10337	0.10337	0.10337	0.10337
	SCA	0.49033211	1.824527	0.090154	0.66195
	GWO	0.05954205	0.266837	0.01197	0.03821

Significant values are in bold.

In addition, the non-parametric Wilcoxon test is applied to the obtained results to determine if the difference between the proposed and each of the other compared methods is significant or not. To reach a suitable decision, there are two hypotheses: null and alternative. The alternative hypothesis supposed that there is no significant difference between the HGS-MPA and the other methods. This occurs when the p-value is less than 0.05. From the p-value in Table 6, it can be noticed that there is a significant difference between the developed HGS-MPA and other tested methods (i.e., HGS, VCA, SCA, GWO, and MPA) among all datasets.

**Table 6:** p-value of Wilcoxon test of comparing HGS-MPA with the relative studies for 20 runs.

	250 W	NedStack PS6	BCS 500 W
HGS	1.920E-07	1.20E-06	1.66E-07
VCA	0.068E-06	6.80E-08	1.92E-07
SCA	0.068E-06	3.99E-06	6.80E-08
GWO	0.068E-06	1.23E-07	2.22E-07
MPA	0.068E-06	6.80E-08	1.66E-07

### Sensitivity analysis

The sensitivity analysis highlights the importance of accurate parameter estimation for achieving reliable predictions with the HGS-MPA model. Small errors in parameter values can lead to significant deviations from the actual system’s behavior. Therefore, it is crucial to use robust optimization algorithms like HGS-MPA and employ accurate measurement techniques to obtain precise parameter estimates. In this part, the effect of sensitivity to small alternate parameters on SSE value was studied as shown in Table 7. The results in Table 7 indicate a high sensitivity of the model parameters to small changes. For most parameters, a small change (e.g., ± 0.1, ± 0.001, ± 1.0E-05) leads to a significant increase in the SSE value. This indicates that the model’s performance is highly dependent on the precise values of these parameters. The high sensitivity of the model to parameter changes is likely due to the inherent nonlinearity of the PEMFC system. Small variations in parameters can have a significant impact on the predicted voltage and current, leading to larger errors.

Hence, Table 7 demonstrates that the HGS-MPA model is highly sensitive to small changes in its parameters. This sensitivity underscores the importance of accurate parameter estimation and highlights the need for robust optimization algorithms and precise measurement techniques to ensure reliable model predictions.

**Table 7:** Sensitivity analysis.

Parameter	Change	250 W	NedStack PS6	BCS 500 W
		SSE	SSE	SSE
ξ_1_	+ 0.1	80.20	462.90	896.16
	-0.1	93.60	8.15E + 03	278.02
ξ_2_	+ 0.001	993.90	2.98E + 05	3.77E + 03
	-0.001	1.04E + 03	3.07E + 04	278.02
ξ_3_	+ 1.0E-05	26.60	5.37E + 03	109.27
	-1.0E-05	19.70	1.43E + 03	535.88
ξ_4_	+ 0.0001	49.80	101.09	802.46
	-0.0001	60.93	1.16E + 04	27.24
λ	+ 1	0.36	2.98E + 03	286.62
	-1	1.04	3.19E + 03	268.52
*R*_c_	+ 0.0001	0.59	3.51E + 03	270.53
	-0.0001	0.41	2.68E + 03	285.61
b	+ 0.01	173.50	3.32E + 03	228.67
	-0.01	154.90	2.85E + 03	334.92
Best SSE		0.3377	0.0117	1.3162

The main advantages of the developed HGS-MPA algorithm are listed as follows:

1. It has better exploration based on HGS’s exploration capability due to many random parameters.
2. It has better exploitation based on MPA’s capability due to the updated strategy based on the skilled predators (Elite matrix) and the best prey.
3. It avoids trapping in local optima due to applying FAD on all agents besides W_1_ and W_2_ of the updating strategy of HGS.

The main limitations of the developed HGS-MPA algorithm are listed as follows:

1. The convergence rate of HGS-MPA needs to be accelerated.
2. HGS-MPA was tested on three PEMFC modules in this work. However, in the future, it needs to be tested on many modules to check its performance.
3. The performance of HGS-MPA was tested for selecting the parameters of PEMFC only, however, it is needed to check its performance on other engineering problems.

## Conclusion and future recommendations

The research paper presents HGS-MPA, a tweaked iteration of the HGS algorithm that integrates the MPA to boost its search capabilities. It aims to extract PEMFC parameters under various operating conditions. The assessment metric utilized involves the summation of squared deviations between the computed and actual voltages of the PEMFC stack. Employing the HGS-MPA algorithm, three datasets—the 250-W stack, BCS 500-W, and NedStack PS6—are subjected to analysis. The results of the developed method are compared with a set of meta-heuristic techniques, including HHO, PO, SMS, ISA, VSA, GWO, SCA, and traditional HGS. From the results, the proposed HGS-MPA has the best results for BCS 500-W, 250-W, and NedStack PS6 are 1.31620, 0.33770, and 0.01174 respectively. Also, the developed HGS-MPA has a convergence rate faster than other methods for the NedStack module, while for the other two datasets, its convergence rate is competitive with MPA and other approaches.

A comparative analysis was performed against related literature and the traditional HGS, employing SSE, standard deviation, and statistical tools like the Wilcoxon test as benchmarks. The findings consistently showcase the superior performance of HGS-MPA across all three datasets for all metrics. This success can be attributed to the balanced nature of HGS-MPA, which combines the effective exploration capabilities of HGS with the efficient exploitation techniques of MPA.

Future suggestions stemming from this study will encompass:

- Application in other fields: The success seen in parameter extraction for PEMFCs suggests the potential for applying HGS-MPA to other fields with similar optimization challenges.
- Algorithmic Fusion: Investigating the fusion of other algorithms or techniques with HGS-MPA such as ensemble methods in machine learning and augmented Lagrangian approach.

might yield new hybrid approaches that could outperform existing methods.

- Enhance the versatility and effectiveness of the HGA-MPA algorithm, future research will focus on adapting it to address the specific characteristics and requirements of various optimization problems. For instance:
- Linear optimization, we will incorporate methods for handling linear constraints, ensuring the algorithm can efficiently explore feasible regions defined by linear inequalities.
- Combinatorial optimization, we aim to introduce strategies tailored to permutation-based problems, including maintaining a list of solutions and efficiently evaluating combinations.
- For multi-objective optimization, we plan to develop a mechanism that balances multiple objectives while maintaining a diverse set of solutions. We will utilize Pareto dominance concepts to guide the search process and allow for the exploration of trade-offs between conflicting objectives.

## Data Availability

The datasets generated during and/or analyzed during the current study are available in another published study (https://doi.org/10.1016/j.renene.2019.08.046).

## Abbreviations

FC: Fuel cell
HGS: Hunger games search
PEMFC: Proton exchange membrane fuel cell
MPA: Marine predator algorithm
SSE: Sum squared error
DE: Differential evolution
GWO: Grey wolf optimizer
FAD: Fish aggregating devices
VSA: Vortex search algorithm
SCA: Sine-cosine algorithm
SMA: Slime Mould algorithm
COA: Coyote optimization algorithm
EOA: Equilibrium optimization algorithm
ASO: Atom search optimization
HHO: Harris Hawk optimization
PO: Political optimizer
NFL: No-free-lunch

## References

[1] Kanani, H., Shams, M., Hasheminasab, M. & Bozorgnezhad, A. Model development and optimization of operating conditions to maximize PEMFC performance by response surface methodology. *Energy Conv. Manag.* **93**, 9–22 (2015).

[2] Ansari, S. A. et al. Modeling and Simulation of a Proton Exchange Membrane Fuel Cell Alongside a Waste Heat Recovery System Based on the Organic Rankine Cycle in MATLAB/SIMULINK Environment. *Sustainability*** 13**, 1218 (2021).

[3] El-Fergany, A. A. Extracting optimal parameters of PEM fuel cells using salp swarm optimizer. *Renew. Energy* **119**, 641–648 (2018).

[4] Wang, L., Husar, A., Zhou, T. & Liu, H. A parametric study of PEM fuel cell performances. *Int. J. Hydrog. Energy* **28**, 1263–1272 (2003).

[5] Famouri, P. & Gemmen, R. S. Electrochemical circuit model of a PEM fuel cell. In *IEEE Power Engineering Society General Meeting (IEEE Cat. No. 03CH37491), 2003*, pp. 1436–1440 (2003).

[6] Correa, J. M., Farret, F. A., Popov, V. A. & Simoes, M. G. Sensitivity analysis of the modeling parameters used in simulation of proton exchange membrane fuel cells. *IEEE Trans. Energy Convers.* **20**, 211–218 (2005).

[7] Feng, Y. et al. *Study on the operating parameter optimization based on the temperature characteristics of fuel cell. Ionics *, 1–12 (2024).

[8] Mann, R. F. et al. Development and application of a generalised steady-state electrochemical model for a PEM fuel cell. *J. Power Sources*. **86**, 173–180 (2000).

[9] Yang, B. et al. Parameter extraction of PEMFC via bayesian regularization neural network based meta-heuristic algorithms. *Energy* **228**, 120592 (2021).

[10] Eid, A., Kamel, S. & Abualigah, L. Marine predators algorithm for optimal allocation of active and reactive power resources in distribution networks. *Neural Comput. Appl.*, pp. 1–29 (2021).

[11] Srinivasulu, G. N., Subrahmanyam, T. & Rao, V. D. *Parametric Sensitivity Analysis of PEM fuel cell Electrochemical Model ed* (Elsevier, 2011).

[12] Yousri, D. et al. Reliable applied objective for identifying simple and detailed photovoltaic models using modern metaheuristics: comparative study. *Energy. Conv. Manag.* **223**, 113279 (2020).

[13] Liu, E. J., Hung, Y. H. & Hong, C. W. Improved metaheuristic optimization algorithm applied to hydrogen fuel cell and photovoltaic cell parameter extraction, *Energies*** 14**, 619 (2021).

[14] Fathy, A. & Rezk, H. Multi-verse optimizer for identifying the optimal parameters of PEMFC model. *Energy* **143**, 634–644 (2018).

[15] Alizadeh, M. & Torabi, F. Precise PEM fuel cell parameter extraction based on a self-consistent model and SCCSA optimization algorithm. *Energy. Conv. Manag.* **229**, 113777 (2021).

[16] Seleem, S. I., Hasanien, H. M. & El-Fergany, A. A. Equilibrium optimizer for parameter extraction of a fuel cell dynamic model. *Renew. Energy* **169**, 117–128 (2021).

[17] Blal, M. et al. Contribution and investigation to compare models parameters of (PEMFC), comprehensives review of fuel cell models and their degradation. *Energy*** 168**, 182–199 (2019).

[18] Gardiner, C. W. *Handbook of Stochastic Methods*vol. 3 (Springer Berlin, 1985).

[19] Abualigah, L., Diabat, A. & Geem, Z. W. A comprehensive survey of the harmony search algorithm in clustering applications. *Appl. Sci.* **10**, 3827 (2020).

[20] Issa, M. Expeditious Covid-19 similarity measure tool based on consolidated SCA algorithm with mutation and opposition operators. *Appl. Soft Comput.* **104**, 107197 (2021). 33642960 10.1016/j.asoc.2021.107197 PMC7895693

[21] Issa, M. & Abd Elaziz, M. Analyzing COVID-19 virus based on enhanced fragmented biological local aligner using improved ions Motion optimization algorithm. *Appl. Soft Comput.*, 106683 (2020). 10.1016/j.asoc.2020.106683 PMC7467904 32901204

[22] Issa, M. Performance optimization of PID Controller based on parameters estimation using Meta-heuristic techniques: a comparative study. In* Metaheuristics in Machine Learning: Theory and Applications*, 691–709 (Springer, 2021).

[23] Issa, M. Enhanced arithmetic optimization algorithm for parameter estimation of PID Controller. *Arab. J. Sci. Eng.*, pp. 1–15 (2022). 10.1007/s13369-022-07136-2 PMC9411853 36042895

[24] Issa, M., Abd Elbaset, A., Hassanien, A. E. & Ziedan, I. PID Controller tuning parameters using Meta-heuristics algorithms: comparative analysis. In* Machine Learning Paradigms: Theory and Application*, 413–430 (Springer, 2019).

[25] Sharma, A. et al. Performance investigation of state-of-the-art metaheuristic techniques for parameter extraction of solar cells/module. *Sci. Rep.* **13**, 11134 (2023). 37429876 10.1038/s41598-023-37824-4 PMC10333343

[26] Lim, W. H. & Sharma, A. Overview of Swarm Intelligence Techniques for Harvesting Solar Energy. In* Recent Advances in Energy Harvesting Technologies*, 161–175 (River, 2023).

[27] Issa, M. & Samn, A. Passive vehicle suspension system optimization using Harris Hawk optimization algorithm. *Math. Comput. Simul.* **191**, 328–345 (2022).

[28] Shams-Shemirani, S., Tavakkoli-Moghaddam, R., Amjadian, A. & Motamedi-Vafa, B. Simulation and process mining in a cross-docking system: a case study. *Int. J. Prod. Res.*, pp. 1–24 (2023).

[29] Issa, M. Digital image watermarking performance improvement using bio-inspired algorithms. In* Advances in Soft Computing and Machine Learning in Image Processing*, 683–698 (Springer, 2018).

[30] Ali, M., El-Hameed, M. & Farahat, M. Effective parameters’ identification for polymer electrolyte membrane fuel cell models using grey wolf optimizer. *Renew. Energy*** 111**, 455–462 (2017).

[31] Gupta, J., Nijhawan, P. & Ganguli, S. Optimal parameter estimation of PEM fuel cell using slime mould algorithm. *Int. J. Energy Res.* (2021).

[32] Abaza, A., El-Sehiemy, R. A., Mahmoud, K., Lehtonen, M. & Darwish, M. M. Optimal Estimation of Proton Exchange Membrane Fuel Cells Parameter Based on Coyote Optimization Algorithm. *Appl. Sci.*** 11**, 2052 (2021).

[33] Jangir, P. et al. Precision parameter estimation in Proton Exchange Membrane Fuel Cells using depth information enhanced Differential Evolution. *Sci. Rep.* **14**, 29591 (2024). 39609578 10.1038/s41598-024-81160-0 PMC11604669

[34] Xuebin, L., Zhao, J., Daiwei, Y., Jun, Z. & Wenjin, Z. Parameter estimation of PEM fuel cells using metaheuristic algorithms, *Measurement*** 237**, 115302 (2024).

[35] Mossa, M. A., Kamel, O. M., Sultan, H. M. & Diab, A. A. Z. Parameter estimation of PEMFC model based on Harris Hawks’ optimization and atom search optimization algorithms. *Neural Comput. Appl.* **33**, 5555–5570 (2021).

[36] Cao, Y., Kou, X., Wu, Y., Jermsittiparsert, K. & Yildizbasi, A. PEM fuel cells model parameter identification based on a new improved fluid search optimization algorithm. *Energy Rep.* **6**, 813–823 (2020).

[37] Sharma, P., Raju, S. & Salgotra, R. An evolutionary multi-algorithm based framework for the parametric estimation of proton exchange membrane fuel cell. *Knowl. Based Syst.* **283**, 111134 (2024).

[38] Fathy, A., Abd Elaziz, M. & Alharbi, A. G. A novel approach based on hybrid vortex search algorithm and differential evolution for identifying the optimal parameters of PEM fuel cell. *Renew. Energy* **146**, 1833–1845 (2020).

[39] Sharma, A., Khan, R. A., Sharma, A., Kashyap, D. & Rajput, S. A Novel opposition-based arithmetic optimization algorithm for parameter extraction of PEM fuel cell. *Electronics*** 10**, 2834 (2021).

[40] Salgotra, R., Sharma, P. & Raju, S. A multi-hybrid algorithm with shrinking population adaptation for constraint engineering design problems. *Comput. Methods Appl. Mech. Eng.* **421**, 116781 (2024).

[41] Khajuria, R., Bukya, M., Lamba, R. & Kumar, R. Optimal parameter extraction of PEM Fuel Cell using a Hybrid Weighted Mean of vectors and Nelder-Mead Simplex Method. *IEEE Access* (2024).

[42] Saad, B., El-Sehiemy, R. A., Hasanien, H. M. & El-Dabah, M. A. Robust parameter estimation of proton exchange membrane fuel cell using Huber loss statistical function. *Energy. Conv. Manag.* **323**, 119231 (2025).

[43] Fathy, A., Abdel Aleem, S. H. & Rezk, H. A novel approach for PEM fuel cell parameter estimation using LSHADE-EpSin optimization algorithm. *Int. J. Energy Res.* **45**, 6922–6942 (2021).

[44] Hachana, O. Accurate PEM fuel cells parameters estimation using hybrid artificial bee colony differential evolution shuffled complex optimizer. *Int. J. Energy Res.* **46**, 6383–6405 (2022).

[45] Li, J. et al. Accurate, efficient and reliable parameter extraction of PEM fuel cells using shuffled multi-simplexes search algorithm. *Energy. Conv. Manag.* **206**, 112501 (2020).

[46] Zheng, J., Xie, Y., Huang, X., Wei, Z. & Taheri, B. Balanced version of Slime Mold Algorithm: a study on PEM fuel cell system parameters identification. *Energy Rep.* **7**, 3199–3209 (2021).

[47] Abdel-Basset, M., Mohamed, R., Elhoseny, M., Chakrabortty, R. K. & Ryan, M. J. An efficient heap-based optimization algorithm for parameters identification of proton exchange membrane fuel cells model: analysis and case studies. *Int. J. Hydrog. Energy*. **46**, 11908–11925 (2021).

[48] Yang, Z., Liu, Q., Zhang, L., Dai, J. & Razmjooy, N. Model parameter estimation of the PEMFCs using improved barnacles mating optimization algorithm, *Energy*** 212**, 118738 (2020).

[49] Houssein, E. H., Helmy, B. E., Rezk, H. & Nassef, A. M. An enhanced Archimedes optimization algorithm based on local escaping operator and orthogonal learning for PEM fuel cell parameter identification. *Eng. Appl. Artif. Intell.* **103**, 104309 (2021).

[50] Rezk, H. et al. Optimal parameter estimation strategy of PEM fuel cell using gradient-based optimizer. *Energy* **239**, 122096 (2022).

[51] Riad, A. J., Hasanien, H. M., Turky, R. A. & Yakout, A. H. Identifying the PEM Fuel Cell Parameters Using Artificial Rabbits Optimization Algorithm, *Sustainability*** 15**, 4625 (2023).

[52] Messaoud, R. B., Midouni, A. & Hajji, S. PEM fuel cell model parameters extraction based on moth-flame optimization. *Chem. Eng. Sci.* **229**, 116100 (2021).

[53] Mujeer, S. A., Chandrasekhar, Y., Kumari, M. S. & Salkuti, S. R. An accurate method for parameter estimation of proton exchange membrane fuel cell using Dandelion optimizer. *Int. J. Emerg. Electr. Power Syst.* **25**, 333–344 (2024).

[54] Gouda, E. A., Kotb, M. F. & El-Fergany, A. A. Jellyfish search algorithm for extracting unknown parameters of PEM fuel cell models: Steady-state performance and analysis, *Energy*** 221**, 119836 (2021).

[55] Han, W., Li, D., Yu, D. & Ebrahimian, H. Optimal parameters of PEM fuel cells using chaotic binary shark smell optimizer. *Energy Sour. Part a Recover. Utilization Environ. Eff.* **45**, 7770–7784 (2023).

[56] Shaheen, A., El-Sehiemy, R., El-Fergany, A. & Ginidi, A. Fuel-cell parameter estimation based on improved gorilla troops technique. *Sci. Rep.* **13**, 8685 (2023). 37248236 10.1038/s41598-023-35581-y PMC10227001

[57] Baradaran Rezaei, H., Amjadian, A., Sebt, M. V., Askari, R. & Gharaei, A. An ensemble method of the machine learning to prognosticate the gastric cancer. *Ann. Oper. Res.* **328**, 151–192 (2023).

[58] Ragab, M., Alshammari, S. M. & Al-Ghamdi, A. S. Modified metaheuristics with Weighted Majority Voting Ensemble Deep Learning Model for Intrusion Detection System. *Comput. Syst. Sci. Eng.*, **47** (2023).

[59] Fonseca, G. B., Nogueira, T. H. & Ravetti, M. G. A hybrid Lagrangian metaheuristic for the cross-docking flow shop scheduling problem. *Eur. J. Oper. Res.* **275**, 139–154 (2019).

[60] Gharaei, A., Amjadian, A., Shavandi, A. & Amjadian, A. An augmented Lagrangian approach with general constraints to solve nonlinear models of the large-scale reliable inventory systems. *J. Comb. Optim.* **45**, 78 (2023).

[61] Yang, Y., Chen, H., Heidari, A. A. & Gandomi, A. H. Hunger games search: visions, conception, implementation, deep analysis, perspectives, and towards performance shifts. *Expert Syst. Appl.* **177**, 114864 (2021).

[62] Faramarzi, A., Heidarinejad, M., Mirjalili, S. & Gandomi, A. H. Marine predators Algorithm: a nature-inspired metaheuristic. *Expert Syst. Appl.* **152**, 113377 (2020).

[63] Sun, Z., Wang, N., Bi, Y. & Srinivasan, D. Parameter identification of PEMFC model based on hybrid adaptive differential evolution algorithm, *Energy*** 90**, 1334–1341 (2015).

[64] Gong, W. & Cai, Z. Parameter optimization of PEMFC model with improved multi-strategy adaptive differential evolution. *Eng. Appl. Artif. Intell.* **27**, 28–40 (2014).

